# Evaluation of ventilation systems as a cooling strategy in dwellings using a validated TRNSYS model

**DOI:** 10.1038/s41598-025-09939-3

**Published:** 2025-07-17

**Authors:** Ícaro C. Vera Alves, Pedro J. Martínez Beltrán, Javier Molina González

**Affiliations:** 1 University Institute for Engineering Research, Miguel Hernández University of Elche, Avda. de la Universidad s/n, Edificio Innova, Elche, Alicante, 03202 Spain; 2 Department of Mechanical and Energy Engineering, Miguel Hernández University of Elche, Avda. de la Universidad s/n, Edificio Innova, Elche, Alicante, 03202 Spain

**Keywords:** TRNSYS, CONTAM, Heat recovery, Ventilative cooling, Nocturnal ventilation, Mechanical engineering, Renewable energy

## Abstract

Free cooling is a passive technique in which ventilation is used to reduce the sensible heat load when outdoor conditions are favourable. This technique is easy to apply in residential buildings, as it can be implemented either naturally or via a mechanical system. However, one should not lose the main purpose of ventilation in buildings: to ensure indoor air quality (IAQ). To assess the applicability of this technique depending on the ventilation system (natural or mechanical), an experimentally validated TRNSYS model of the detached single-family dwelling La Casa de la Tierra, coupled with a natural ventilation model in CONTAM, was applied in case studies. As a novelty, both systems are integrated into the legislative framework of minimum ventilation requirements to ensure IAQ and comfort. The results indicated that combining both systems during summer days and nights provides a much more attractive solution than relying on a single system throughout the year. Furthermore, in addition to its straightforward implementation, the proposed solution ensures IAQ, with a reduction in ventilation energy consumption of up to 46.2% in summer and allows 6.3 times more climatic cooling potential to be harnessed.

## Introduction

Europe has set the goal of achieving climate neutrality by 2050 and aims to be the first continent to do so. Building decarbonisation is one of the measures implemented by the governments of European countries to achieve this goal. In Europe, buildings are responsible for 40% of all energy consumption and 36% of all greenhouse gas emissions^1^. In addition, it is estimated that 75% of the building stock is not efficient and that 85–95% of existing buildings will still exist in 2050. In the residential sector, approximately 80% of the energy consumed is used for heating, cooling and domestic hot water purposes. On a more local level, in Spain, dwellings were responsible for 18% of the final energy consumption in 2023^2^.

Within this context, the Energy Performance of Buildings Directive (EPBD)^3^ and the concept of nearly zero-energy buildings (nZEB) emerged, which promoted improvements in the energy efficiency of buildings in the European Union and whose transposition at the national level was realized through the Basic Document on Energy Saving of the Spanish Technical Building Code (CTE DB-HE)^4^.

Among the various existing solutions for reducing energy consumption are passive systems. These systems, which have been around since ancient times, offer sustainable alternatives to conventional techniques^5^. Free cooling represents a group of techniques that make it possible to reduce the cooling demand by harnessing the outside climate. The efficiency of these techniques depends on the climate in the area where they are implemented and must be considered during the design phase of buildings.

Free cooling, as a passive technique in dwellings, is usually associated with the night cooling technique^5^which consists of using outside air to reduce the cooling demand. This is done when the outdoor air temperature is lower than the indoor air temperature, and generally, in dwellings, this condition occurs mostly during nighttime hours. If a building possesses a sufficient thermal mass, fresh air can cool the mass of the dwelling, which can serve as a heat sink during the day, absorbing heat gains and delaying the increase in the indoor temperature^6–8^. The latter translates directly into energy savings.

The efficiency of nighttime ventilation depends on or is related to multiple factors. Blondeau et al.^6^ classified factors into three main groups: climatic parameters, parameters associated with the building and technical parameters.

The climate itself is a crucial indicator, as understanding or estimating the thermal behaviour of a building enables the assessment of the potential of this technique. According to these authors, the most relevant climatic parameter is the mean outdoor temperature. There are other indicators, such as the minimum outdoor temperature (T_min_) and the daily temperature range (DTR), which indicate the difference between the daily maximum and minimum temperatures. Lower T_min_, higher DTR and of course the presence of wind at night indicate that a climate has a high cooling potential^9–11^.

Artmann et al.^12^ introduced the concept of the climate cooling potential (CCP) to evaluate the nighttime cooling potential in a given climate zone coupled with a harmonic model of the indoor temperature of a building free-floating around comfortable conditions. The metric proposed by these authors is based on the degree hours, and the unit is Kelvin-hour per night. It has been estimated that approximately 80 Kh/night is needed to dissipate 50 W/m^2^ of heat from a given building for an occupancy time of 8 h. According to the same study, regions in northern Spain offer between 60 and 140 Kh/night, whereas regions farther south provide less than 60 Kh/night.

Subsequently, Campaniço et al.^13^ improved the CCP concept by introducing the useful cooling potential (UCP). This new indicator allows comparison of the cooling potential with the cooling demand of a building by expressing the potential in more familiar units, i.e., in kWh. In a later work, these authors evaluated the potential of ventilation cooling techniques for the entire Iberian Peninsula. They concluded that there is clear asymmetry between the northern and southern regions, with the former exhibiting the greatest cooling potential. In fact, they estimated a potential above 1 kWh/m³ for ventilated buildings during the cooling season for the Iberian Peninsula^14^.

Among the aspects related to the building, one of the most influential is the thermal mass. This parameter, which is different for each material that composes the building, is expressed as the product of the density of the material and its specific heat. Depending on whether the element is in contact with the outside or inside air, it is classified as external thermal mass or internal thermal mass^10^. In general terms, a lightweight building responds quickly to changes in outside temperature, while a building with sufficient thermal mass allows energy from the thermal load to be absorbed slowly and then released at night, attenuating indoor temperature fluctuations and reducing the indoor temperature peak, resulting in improved thermal comfort.

Technical parameters are associated with the type of ventilation or aspects related to the means of estimating the efficiency of the system. In the first group, i.e., the type of ventilation, nighttime ventilation can be achieved by mechanical or natural means. On the one hand, mechanical means always ensure a ventilation flow rate, which, as will be determined later, can be used to justify the indoor air quality (IAQ). However, these systems consume energy and do not exhibit passive characteristic. In fact, the main difficulty in using outside air for cooling via mechanical means is energy consumption^15^. On the other hand, natural ventilation (the stack effect and external wind) does not provide a constant airflow, but it will always be beneficial if the outside air is cooler, as there is no energy consumption. With respect to the second group of technical parameters, the prediction of thermal comfort, i.e., estimating how the indoor temperature will fluctuate, is closely related to the indoor convection algorithm employed in simulations^8^.

Regarding energy benefits, various authors reported their results as reductions in the cooling demand or cooling energy consumption. Blondeau et al.^6^ estimated reductions in cooling energy consumption between 12% and 54% for setpoint temperatures ranging from 22 to 26 °C, indicating that mechanical cooling of buildings during the daytime reduces the efficiency of nighttime cooling. Santamouris et al.^16^ estimated that night ventilation can reduce the cooling demand of residential buildings by up to 40 kWh/m^2^-year, with an average reduction of approximately 12 kWh/m^2^-year for buildings with cooling loads between 50 and 60 kWh/m^2^-year. Assuming a constant seasonal efficiency ratio (SEER), these values translate to an average reduction in cooling energy consumption of 20–24%. Darmanis et al.^17^ reported reductions in cooling energy consumption between 15% and 27% with the use of nighttime ventilation. Stasi R et al.^18^ reported similar savings, between 1.9 and 21.8% savings in cooling energy consumption. Additionally, other authors reported a reduction of 0.31 kWh/m^2^-day in the cooling demand with a daily amplitude of 6 K^19^which could amount to approximately 24 kWh/m^2^-year during the summer period.

Nighttime ventilation also exhibits several disadvantages or limitations. Solgi et al.^8^ highlighted the difficulty of manual control (in the case of natural ventilation), the presence of insects, security, rain and dust as barriers. Additionally, large cities or urban centres could exhibit an effect known as the urban heat island phenomenon that could reduce the effectiveness of this technique. Furthermore, its dependence on climate means that for future climates with higher temperatures and longer summer seasons the potential of this technique will be reduced^9,11,20,21^ and a hybrid approach or coupled with another passive strategy would be of interest.

In addition to the above disadvantages, there are acoustics and the need to minimise ventilation flow during the day. Ideally, ventilation would only occur at night, thus preventing the air during the day from serving as a thermal load and allowing the building to absorb more energy. However, this is not possible because the indoor air quality (IAQ) must be ensured.

### IAQ requirements in Spanish dwellings

Most dwellings in Spain are equipped with natural ventilation systems containing pipes or ducts that naturally extract air from humid rooms, and fresh air enters dry rooms through infiltration. These systems suffer drawbacks such as flow reversal or insufficient ventilation flow rates. García-Ortega and Linares-Alemparte^22^ concluded that 50% of the studied dwellings with natural ventilation systems do not meet the IAQ requirements established by the CTE. In winter, bedrooms exhibited the worst IAQ due to the greater compartmentalisation. In fact, according to these authors, occupants implement measures in winter that adversely affect the IAQ (closing windows and interior doors), with the opposite behaviour in summer.

In Spain, the CTE^4^ includes ventilation requirements in the Basic Document on Hygiene CTE DB-HS/3. For any solution, whether hybrid (natural and mechanical) or purely mechanical, the following requirements must be met by via design conditions:

- For habitable spaces, the annual average CO_2_ concentration should be less than 900 ppm.
- The annual cumulative CO_2_ concentration exceeding 1600 ppm should be less than 500 000 ppm-h.
- The minimum airflow rate should at least 1.5 L/s per habitable space during periods of non-occupancy.

The above requirements are considered satisfied if a ventilation system is established that provides a constant airflow rate, established in accordance with CTE DB-HS/3, depending on the type of dwelling and habitable spaces. In Spain, a typical single-family dwelling has as spaces: three bedrooms, kitchen, living room plus toilets/bathrooms^22^. This, in the case of a constant flow ventilation system, corresponds to a ventilation flow rate of around 120 m^3^/h according to Spanish legislation.

In a study in assistance to the Long-Term Strategy for Energy Rehabilitation in the Building Sector in Spain (ERESEE)^23^a segmentation of the residential housing stock in Spain into typological clusters was conducted. This study shows that a typical single-family dwelling in Spain has an average surface area of around 120 m^2^ which at a height of 2.5 m represents 300 m^3^. This, together with the ventilation flow rate mentioned above, allows us to estimate that a single-family dwelling in Spain would need around 0.4 h^−1^ of air changes per hour (ACH) of ventilation.

Generalising to a European level, according to a study by Zukowska D. et al.^24^ in different European countries, mechanical ventilation is not expressly required in any of them. However, as in Spain, these countries have minimum ventilation requirements which are imposed as ventilation flow rates depending on floor area, occupancy or number of rooms. Similarly, fresh air is provided in dry rooms and stale air is extracted from humid rooms.

Mechanical ventilation systems with heat recovery (MHRV) have become increasingly attractive in order to meet the requirements of low energy consumption in buildings. Furthermore, this active system combined with passive systems (a strategy permitted by Spanish regulations) helps reduce heat losses^25^. Another advantage of these systems is the possibility of bypassing the heat recovery depending on the outdoor conditions and indoor needs, which positions them as a viable solution to take advantage of free cooling and maintaining the IAQ.

Ventilation solutions should be simple and easy for the users to operate, with the residential environment as the easiest to design^15^. Authors such as Xiang and Li^19^ concluded that human behaviour is the most important factor in addition to climate when the start and operation times of nighttime ventilation are considered. However, if indoor conditions remain uncomfortable for a prolonged period, people take action to regulate their comfort. Domínguez-Amarillo et al.^26^in a survey conducted among residents in southern regions of Spain, reported that most users employ passive control measures before switching on air-conditioning equipment. According to these authors, in summer, users open doors and windows or switch on auxiliary fans before employing cooling equipment, a practice that is almost universal^15^.

### Aims of this work

Given the need to ensure the IAQ in buildings, the implementation of at least one unit to guarantee airflow rates has become mandatory in new buildings. However, Spanish regulations still allow for the use of natural ventilation systems as long as the IAQ is ensured. Mechanical systems, although they involve additional energy consumption, also make it possible to harness the potential of ventilation for cooling. A more complete solution consists of a heat recovery system, which, in addition to offering significant energy savings in winter, allows the nighttime cooling potential to be utilised during the summer months via bypass systems. During the day, energy is then recovered by precooling the incoming air. This solution, which is initially considered optimal throughout the year, requires a more exhaustive analysis in summer owing to its energy consumption to determine during which periods it is viable in terms of energy consumption or if, on the contrary, natural ventilation can ensure the IAQ and reduce energy consumption.

This study aimed to answer the above question and presents as a novelty an integrated approach with local regulatory requirements in terms of ensuring IAQ. For this purpose, an experimentally validated energy model of La Casa de la Tierra, a detached single-family dwelling with a sophisticated natural ventilation system, was established in TRNSYS^27^ and coupled with CONTAM^28^. In addition, a mechanical ventilation system with a sensible heat recovery system was installed in the dwelling, enabling a case study between natural and mechanical ventilation. Since the main objective was to evaluate the ability of ventilation to reduce indoor temperatures and since the mechanical ventilation system only incorporates sensible heat recovery, the analysis of the ventilation thermal load focused solely on the sensible component.

## Methods

To achieve the objectives of this study, the following tools were used: a reference building with the ability to implement and compare mechanical and natural ventilation modes, software that allows energy simulation of buildings and the meteorological year at the building location.

### Reference building

**Fig. 1:**
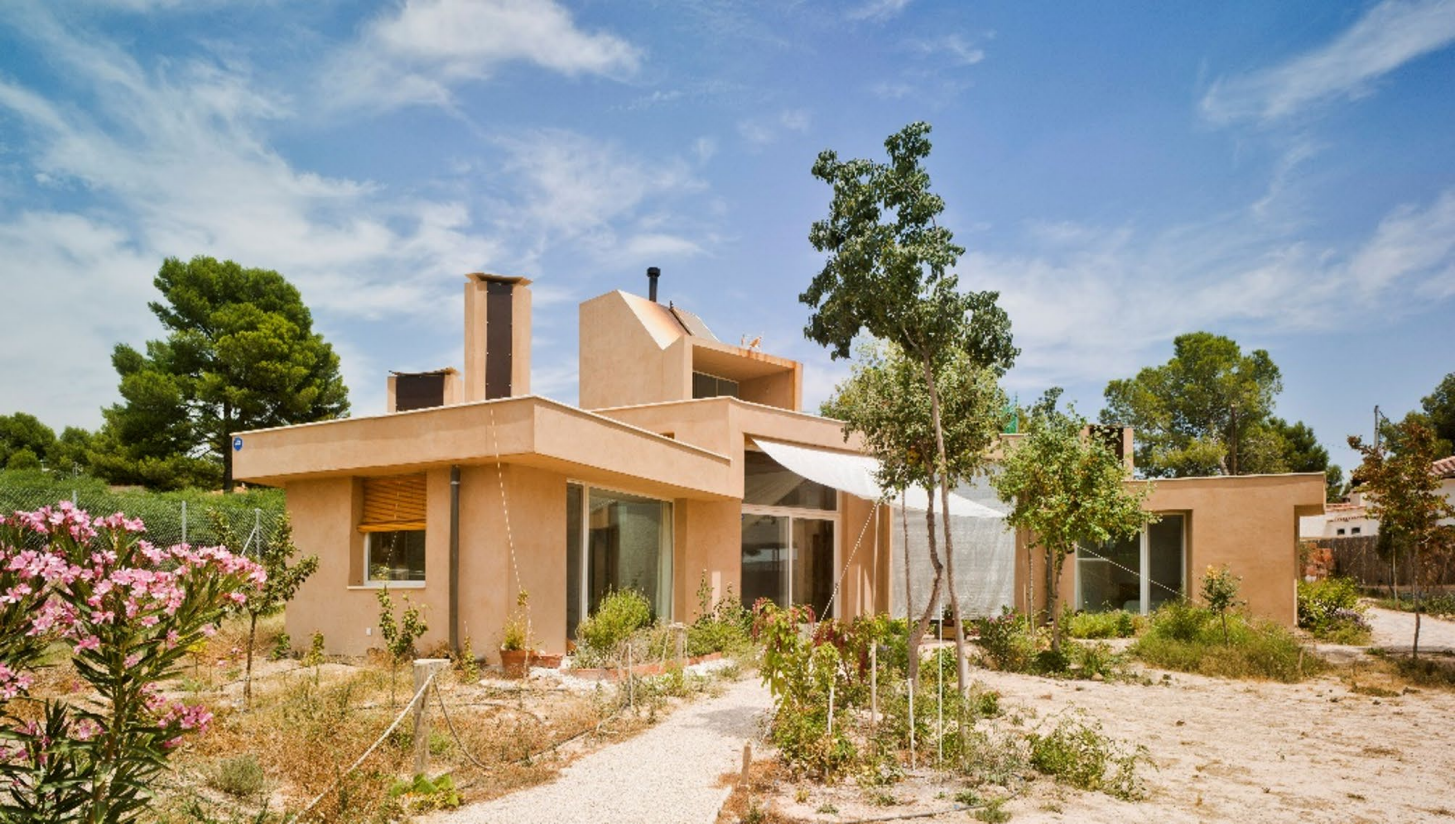
Southern façade of La Casa de la Tierra^29^.

**Fig. 2:**
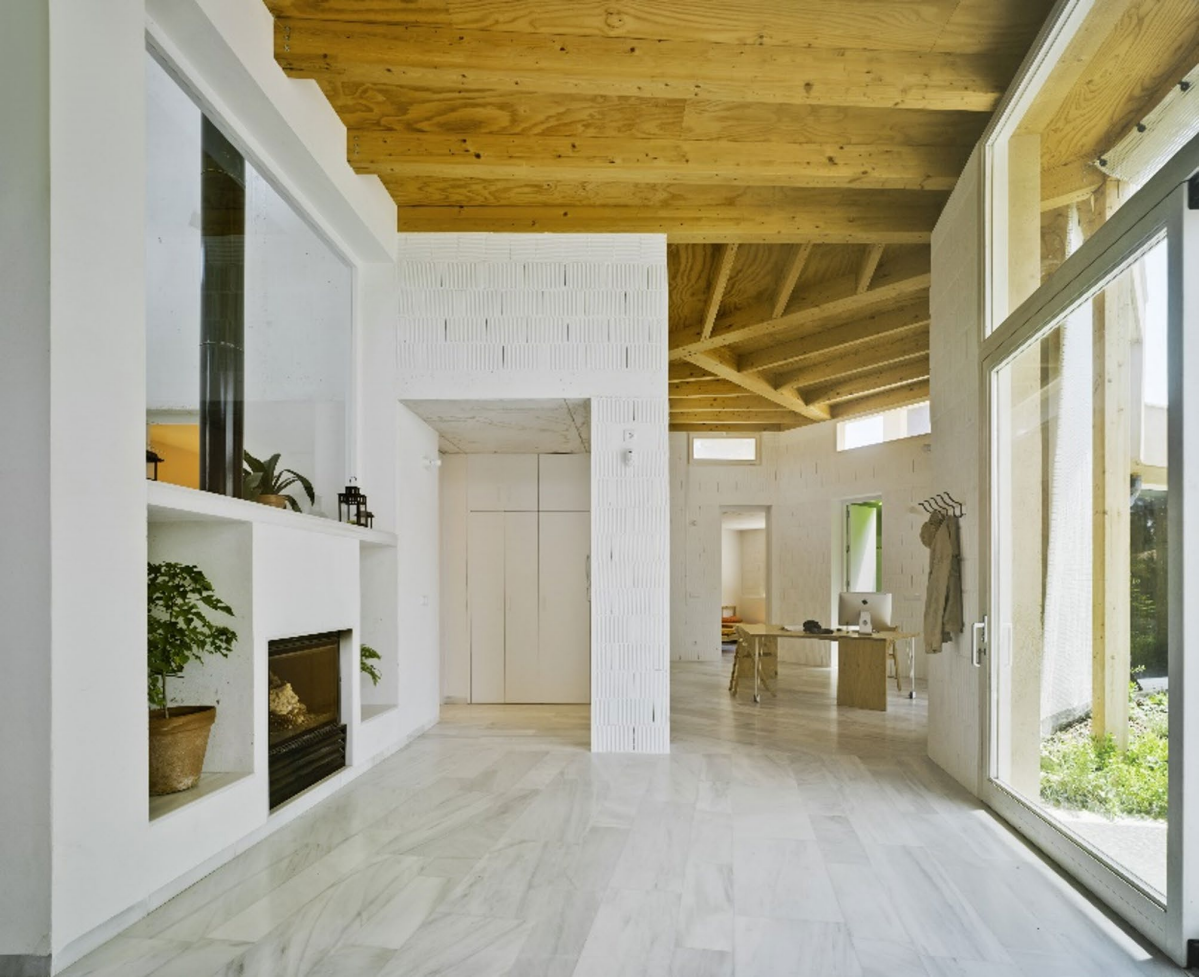
Interior view of La Casa de la Tierra.

The reference building is La Casa de la Tierra, which is a detached single-family dwelling built between 2011 and 2012. Figures 1 and 2 show photographs of its southern façade and interior, respectively. The dwelling is located in Los Valientes (Murcia), a calm residential municipality surrounded by a natural environment.

La Casa de la Tierra was conceived as a bioclimatic house and provides a series of passive strategies aimed at reducing the energy demand. Its main structure is composed of lightweight clay load-bearing walls insulated on the outside with the northern façade partially buried. The interior finish is a layer of white paint. The roof exhibits the same philosophy, with two components: a green roof on a concrete slab and gravel roof on a wooden slab.

These envelopes have their main thermal mass element (lightweight clay, concrete, and wood) in contact with the air inside the dwelling (easily observable in Fig. 2), which provides the building with internal thermal mass. This house has been the subject of previous research^29,30^ and Table 1 shows the thermal-physical building properties.

**Table 1:** Thermal-physical Building properties.

Element	U (W/m^2^-K)	Area (m^2^)		
**Exterior wall**	0.55	128.4		
**Layers**	**t (m)**	**k (W/m-K)**	**ρ(kg/m** ^**3**^ **)**	**c (kJ/kg-K)**
Lime mortar	0.01	0.550	1250	1.00
Insulation mortar	0.04	0.042	150	1.00
Lightweight clay block	0.29	0.421	920	1.40

Element	U (W/m^2^-K)	Area (m^2^)		
**Ground floor**	0.84	150.7		
**Layers**	**t (m)**	**k (W/m-K)**	**ρ(kg/m** ^**3**^ **)**	**c (kJ/kg-K)**
Stone	0.02	3.500	2700	1.00
Cement mortar	0.05	0.550	1250	1.00
EPS	0.02	0.036	-	-
Insulation mortar	0.08	0.410	1000	1.00
Reinforced concrete	0.06	2.500	2500	1.00

Element	U (W/m^2^-K)	Area (m^2^)		
**Gravel roof**	0.23	120.8		
**Layers**	**t (m)**	**k (W/m-K)**	**ρ(kg/m** ^**3**^ **)**	**c (kJ/kg-K)**
Gravel	0.12	2.000	1950	1.05
Geotextile	0.003	0.050	-	-
XPS	0.04	0.034	-	-
Geotextile	0.003	0.050	-	-
EPDM sheet	0.002	0.250	-	-
Insulation mortar	0.08	0.410	1000	1.00
Breathable sheet	0.001	0.220	-	-
Plywood roofing board	0.016	0.210	800	1.60
MW	0.09	0.036	-	-
Plywood roofing board	0.016	0.210	800	1.60

Element	U (W/m^2^-K)	Area (m^2^)		
**Green roof**	0.47	22.4		
**Layers**	**t (m)**	**k (W/m-K)**	**ρ(kg/m** ^**3**^ **)**	**c (kJ/kg-K)**
Vegetation	0.02	0.520	2050	1.84
Geotextile	0.003	0.050	-	-
Drainage sheet (HDPE)	0.003	0.500	-	-
XPS	0.04	0.034	-	-
Geotextile	0.003	0.050	-	-
EPDM sheet	0.002	0.250	-	-
Insulation mortar	0.08	0.410	1000	1.00
Reinforced concrete	0.2	2.500	2500	1.00

Element	U (W/m^2^-K)	Area (m^2^)		
Fenestration		62.9		
Frame	2.2			
Glazing 1 (5 + 5/12/6)	2.8			
Glazing 2 (5 + 5/12/Planitherm XN 6)	1.6			

#### Ventilation system

**Fig. 3:**
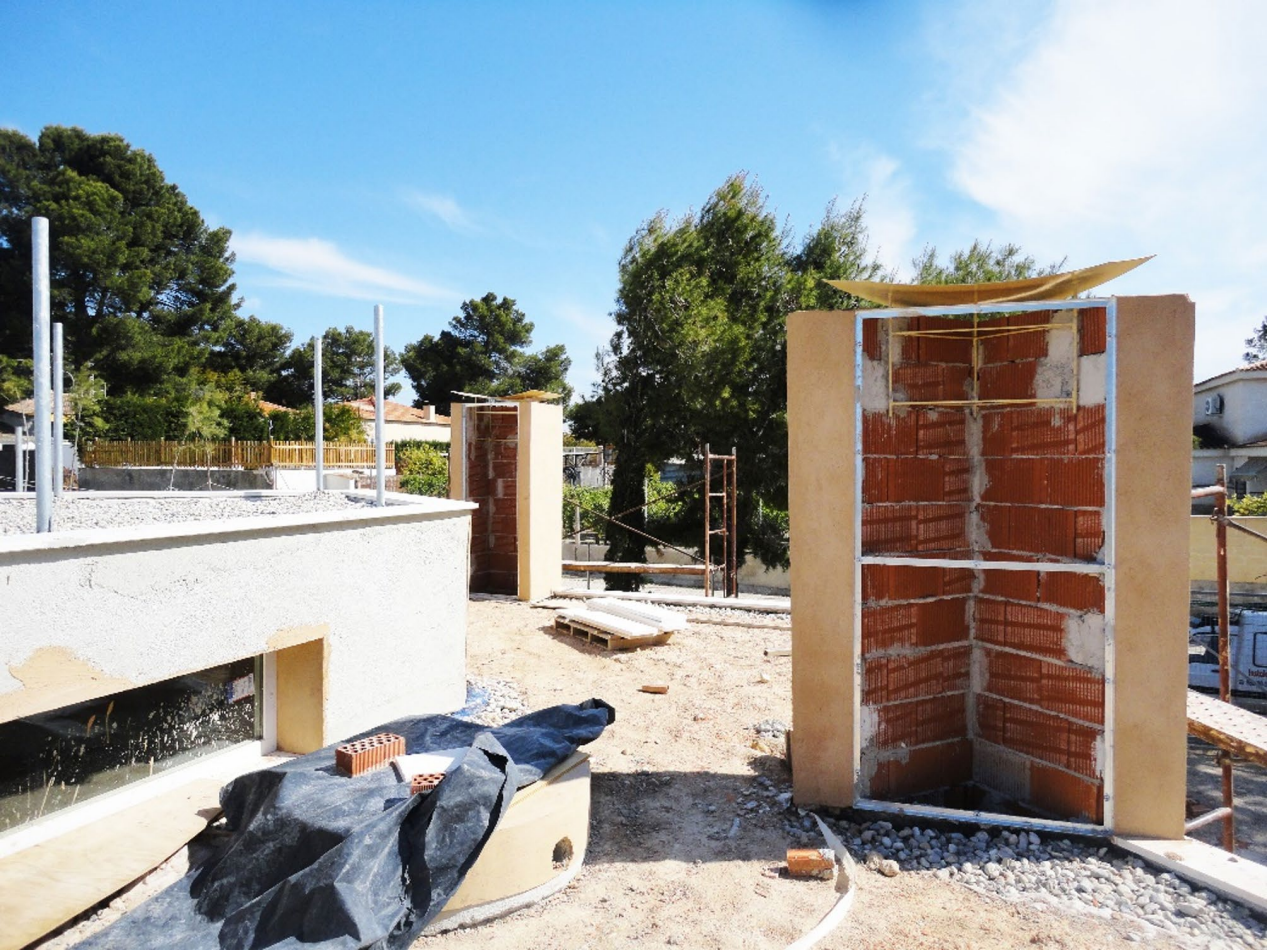
Chimney 4 at the construction stage.

**Fig. 4:**
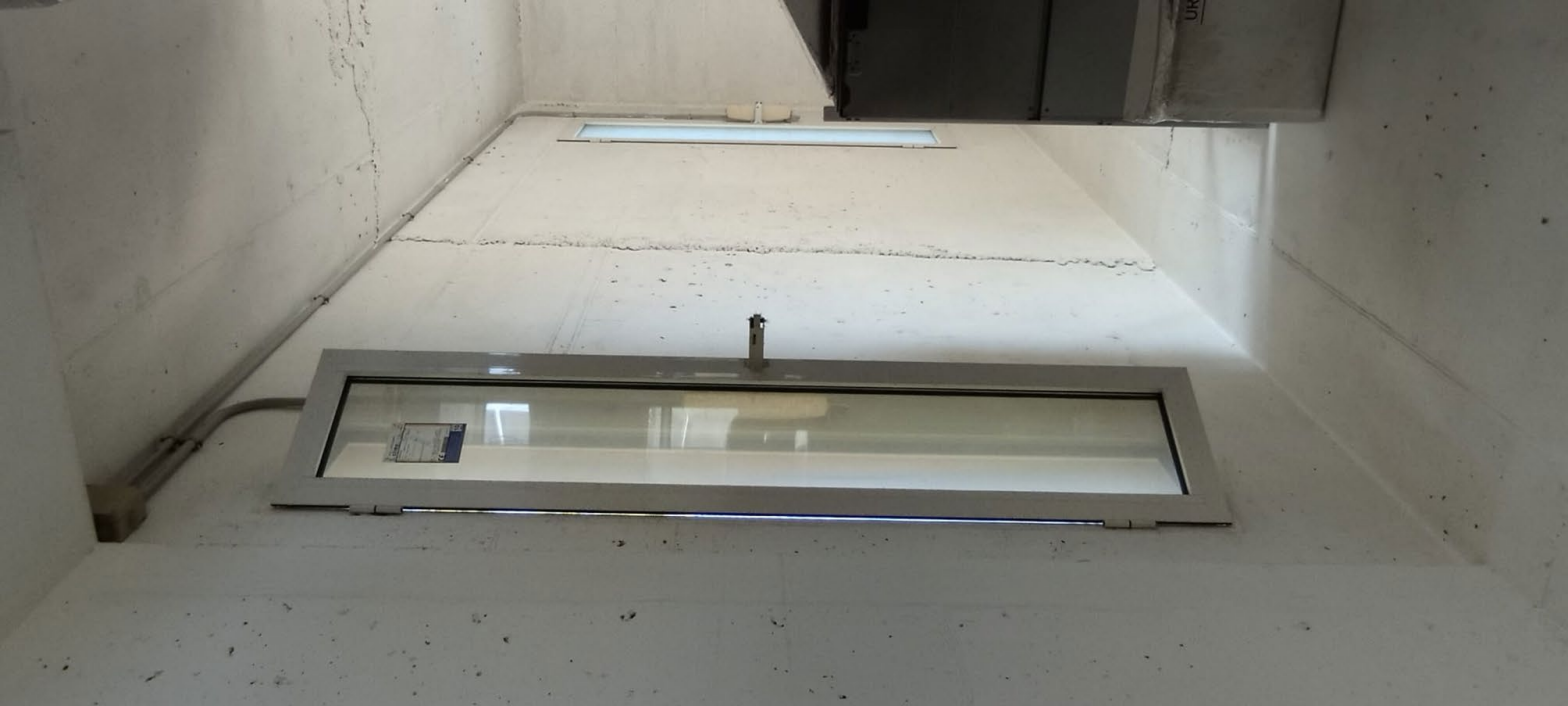
Interior view of the wind tower inlet.

La Casa de la Tierra is of great interest because it features a natural ventilation system consisting of four solar chimneys and a wind tower. The solar chimneys are composed of steel sheets that function as heat absorbers. Their normal operation consists of air extraction via the stack effect during daytime hours and fresh air entering the house through the wind tower and open windows. During summer nights, between 9 p.m. and 9 a.m., the users open windows in the southern façade to increase the airflow rates. Figure 3 shows a photograph of chimney 4 at the construction stage, and Fig. 4 shows an interior view of the wind tower.

The wind tower contains only north-facing air inlets. These inlets are casement-type windows (Fig. 4). In parallel with this system, a mechanical ventilation unit with sensible heat recovery (MHRV), model ENY-SP-180, was recently installed. This unit features a bypass system that allows direct ventilation depending on the outdoor air conditions. Table 2 presents information on the MHRV datasheet.

**Table 2:** MHRV datasheet.

Model	ENY-SP-180
Maximum flow rate	180 m^3^/h
Reference flow rate	130 m^3^/h
Power supply at reference flow rate	23 W
Specific fan power (SFP)	0.174 W/(m^3^/h)
Duct connection	DN125

In total there are 150 m^2^ of interior space distributed in: living room, kitchen, office, three bedrooms, two bathrooms and the wind tower, resulting in 421 m^3^ of habitable volume. According to the requirements of the CTE DB-HS/3, which are determined solely by the number and type of rooms, this dwelling requires a ventilation rate of at least 130 m^3^/h or 0.31 h^−1^. Extending this configuration to a European level, according to the work of Zukowska D. et al.^24^ if La Casa de la Tierra existed in any of the countries studied, the ventilation rate in ACH would be similar to that required in Spain (Table 3).

**Table 3:** Minimum ventilation rates for La Casa de La Tierra in other European countries.

Country	Austria	Denmark	Estonia	France	Norway	UK
**ACH**	0.33	0.19	0.32	0.25	0.36	0.19

The latter presents La Casa de la Tierra as the ideal environment to carry out this type of study, not only because of its ventilation systems, but also because it represents, for the purposes of ventilation needs, a typical single-family dwelling in the current housing stock both in Spain (Sect. IAQ requirements Spanish dwellings) and in some regions of Europe (Table 3).

#### Building energy model

**Fig. 5:**
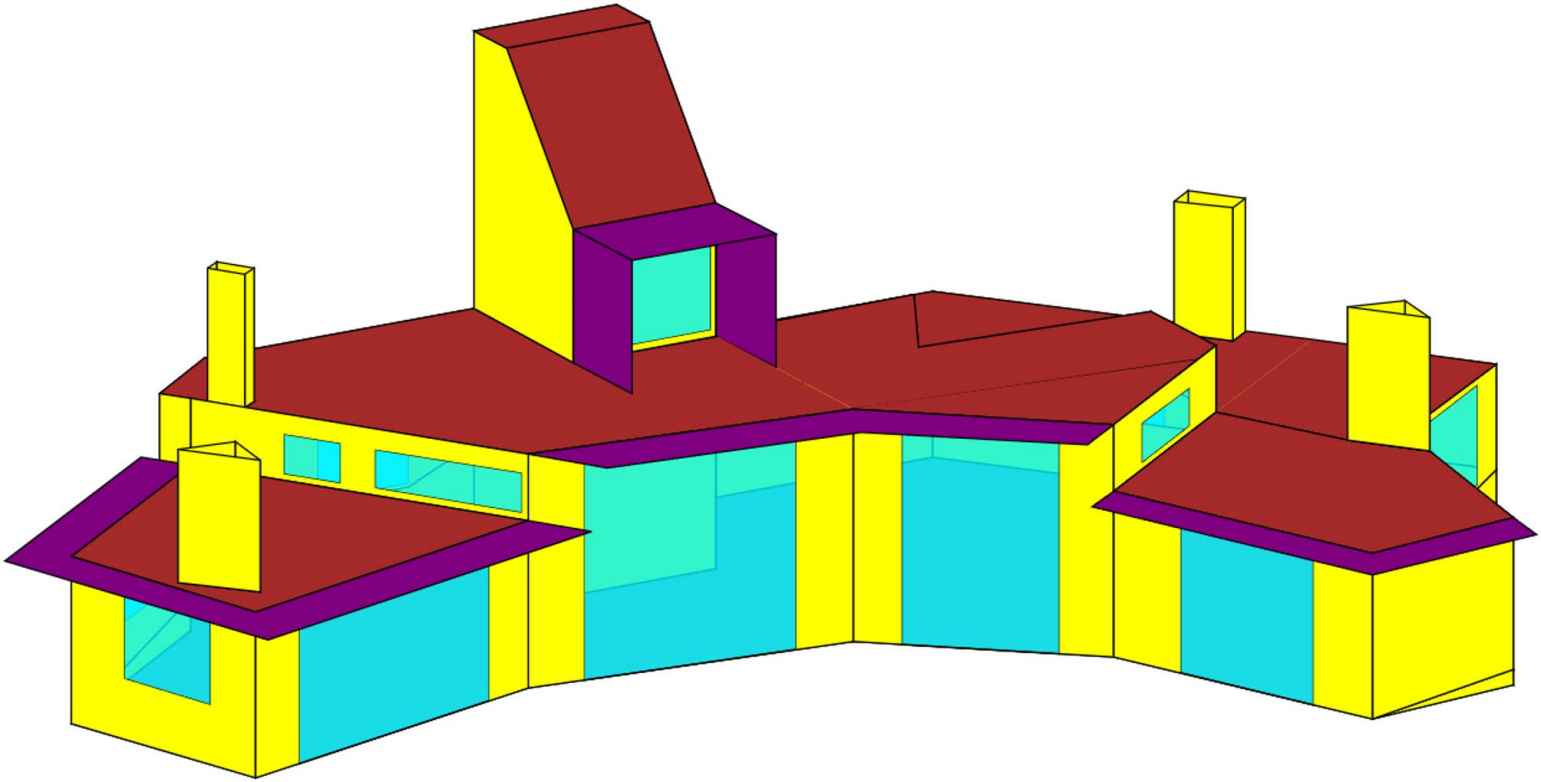
Model of La Casa de la Tierra in TRNBuild.

The energy model of the building was developed in TRNSYS^27^ using the Type56 Multizone Building, which allows the modelling of buildings via thermal zones (multizone). Figure 5 shows the geometrical result of the model. A total of six nodes or thermal zones were modelled:

- Node {1}: Living space zone of the dwelling except for the wind tower.
- Node {2}: Wind tower.
- Nodes {3–6}: Solar chimneys.

#### Natural ventilation model

The natural ventilation model is an airflow network model (AFN). These models consist of nodes and links. The nodes are points associated with the hygrothermal properties of air, and the links are junctions between two or more nodes. At each link, the airflow is assumed to be governed by Bernoulli’s equation (Eq. (1)).

1 $$\:\varDelta\:P=\left({P}_{i}+\frac{\rho\:{V}_{i}^{2}}{2}\right)-\left({P}_{j}+\frac{\rho\:{V}_{j}^{2}}{2}\right)+\rho\:g({z}_{i}-{z}_{j})$$

where:

- $\:\varDelta\:P$: the total pressure drop between nodes i and j;
- $\:{P}_{i}\:\text{a}\text{n}\text{d}\:{P}_{j}$: the static pressure at the inlet and outlet of the link;
- $\:{V}_{i}\:\text{a}\text{n}\text{d}\:{V}_{j}$: the speed of entry and exit of the link;
- $\:\rho\:$: the air density;
- $\:g:$ the acceleration of gravity; and
- $\:{z}_{i}\:\text{a}\text{n}\text{d}\:{z}_{j}:$ the elevations of the inlet and outlet, respectively.

Then, the airflow through each element can be modelled by potential or quadratic flow (Eq. (2)).

2 $$\:Q={C}_{d}A\sqrt{\frac{2\varDelta\:P}{\rho\:}}$$

where:

- $\:Q$: the volumetric airflow rate between the nodes over a link;
- $\:{C}_{d}$: the discharge coefficient; and
- $\:A$: the orifice opening area.

This study presents a novel natural ventilation model of La Casa de la Tierra developed in CONTAM^28^ using Type97. This model represents a system composed of solar chimneys and a wind tower. CONTAM^28^ is an indoor air quality and multizone ventilation analysis program. In a graphical way, this program allows us to define thermal zones and to establish the equations governing natural ventilation in AFN models.

**Fig. 6:**
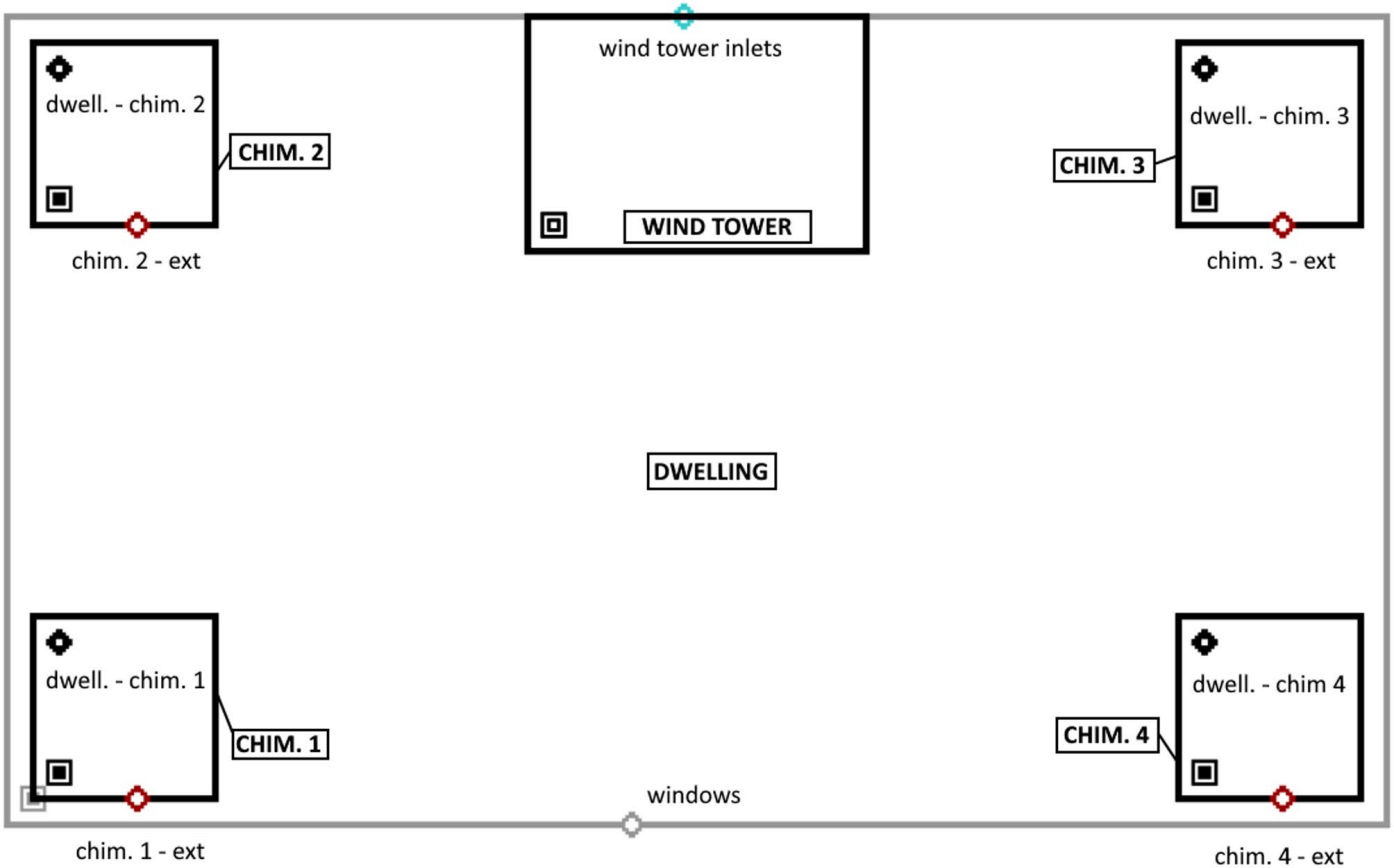
CONTAM SketchPad.

The main advantage of using CONTAM^28^compared to previous models, is the speed of calculation in onion-type simulations, a method also applied via Type97^31^. This allows studies to be conducted for 8760 h of the year with less computational time. Figure 6 shows the model developed in CONTAM. Its design interface, called SketchPad, allows the definition of the zones that compose the model, in this case the same nodes as the thermal model (defined in sect. "Building energy model"). These nodes take as properties the height of the zone and its volume, in Fig. 6 this is represented by rectangular shapes. The larger rectangle represents the living space node of the dwelling which in turn is connected to four smaller rectangles representing the solar chimneys (located one in each corner) and a rectangle in the upper centre representing the wind tower.

The connections between zones (or between zone-outdoors) are made by means of airflow elements which are represented in the SketchPad by diamond shapes. For this model, quadratic flow elements were used (Eq. 2), so it was necessary to define the discharge coefficients (*C*_*d*_) and the orifice area (*A*). All these parameters are detailed in Table 4.

**Table 4:** CONTAM airflow path parameters^29^.

Airflow path	Elevation (m)	Cd (-)	A (m^2^)
Outdoor to tower	4.65	0.40	0.95
Dwelling to chimney 1	2.50	1.00	0.01
Dwelling to chimney 2	2.50	0.80	0.02
Dwelling to chimney 3	2.50	1.00	0.01
Dwelling to chimney 4	2.50	1.00	0.01
Chimney 1 to outdoors	4.50	0.60	0.32
Chimney 2 to outdoors	4.50	0.60	0.06
Chimney 3 to outdoors	4.50	0.60	0.30
Chimney 4 to outdoors	4.50	0.60	0.22
Dwelling to outdoors	1.00	1.00	5.80

Type97 inputs are the temperatures of each thermal zone (information coming directly from Type56), outside wind direction and speed. The outputs of Type97 are defined as infiltrations for flows coming from outside and coupling airflows for flows coming from other nodes/thermal zones. The final result (temperatures and airflow rates) is obtained by convergence of the two models.

#### MHRV system

This system was modelled using Type147 (variable-flow fan) and Type91 (sensible heat recovery). The control for bypass was performed using Type11c (mixing valve), Type166 (thermostat) and Type165 (differential on/off control) to evaluate the indoor–outdoor temperature difference. The energy consumption of the fans was estimated by the specific fan power parameter (SFP) declared by the manufacturer, which is 0.174 Wh/m^3^. The system is operated at the minimum flow rate that ensures the IAQ, in this case, 130 m^3^/h.

#### Experimental campaign

Two experimental campaigns were performed in the dwelling. The first campaign, conducted at the end of July, aimed to characterise the thermal behaviour of the dwelling and validate the natural ventilation model. For this purpose, the indoor and outdoor temperatures, as well as the air velocity at the entrances of the four chimneys, were measured via sensors calibrated in the laboratory. Additionally, CO_2_ levels inside the dwelling were measured to determine the indoor air quality.

A second campaign was subsequently conducted at the end of October, focusing exclusively on the MHRV system. This campaign aimed to measure the sensible effectiveness ($\:\epsilon\:$) of the system according to Eq. (3) and validate its bypass operation. Temperatures were measured at the inlets of the outdoor air intake, fresh air supply, and stale air return.

3 $$\:\epsilon\:=\frac{T\left[2\right]-T\left[3\right]}{T\left[1\right]-T\left[3\right]}$$

where:

- $\:\epsilon\:$: the sensible effectiveness;
- $\:T\left[1\right]$: the temperature at the stale air return (°C);
- $\:T\left[2\right]$: the temperature at the fresh air outlet (°C); and
- $\:T\left[3\right]$: the temperature at the outdoor air intake (°C).

**Fig. 7:**
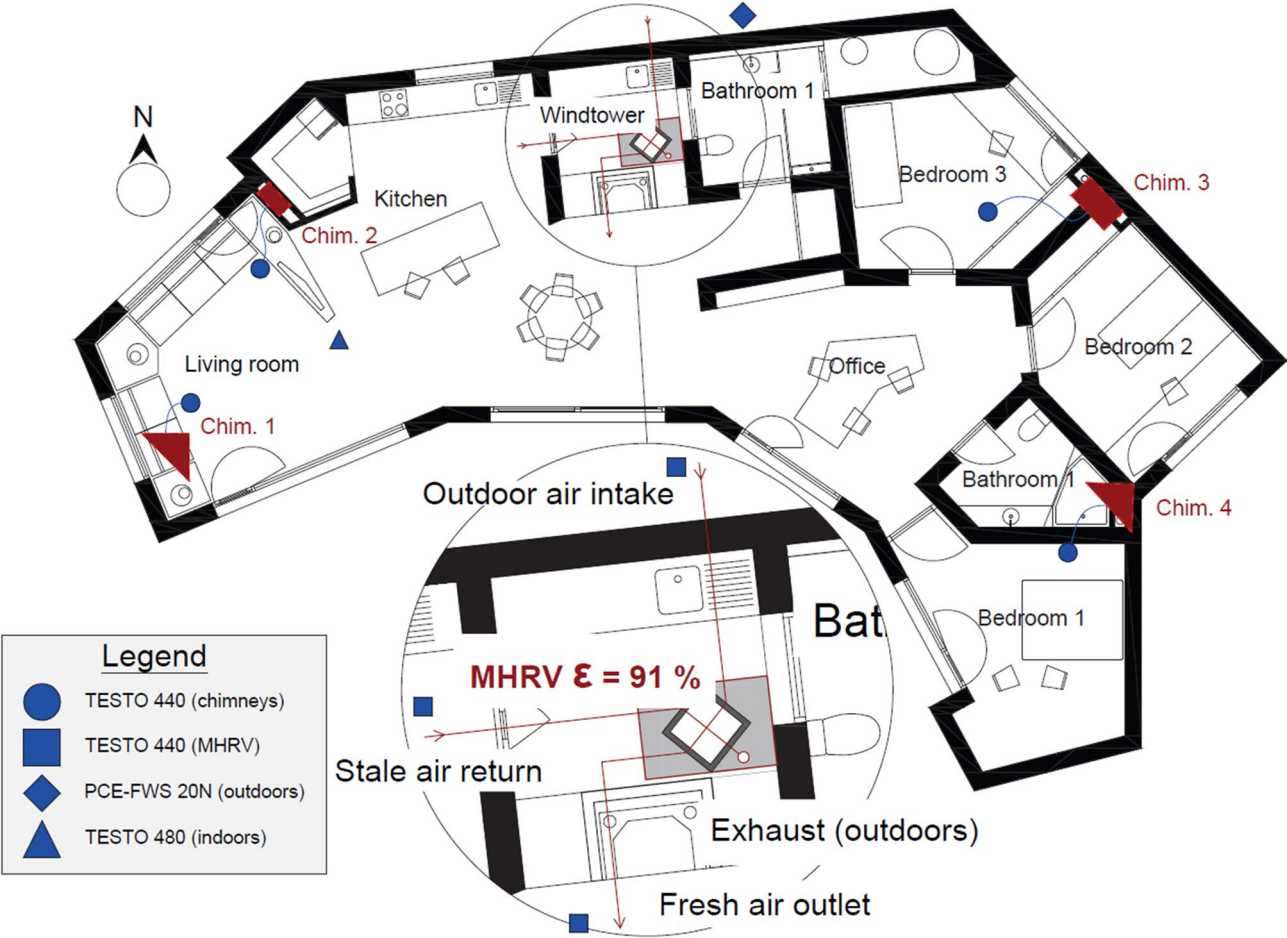
Floor plan of the dwelling and measurement points.

**Fig. 8:**
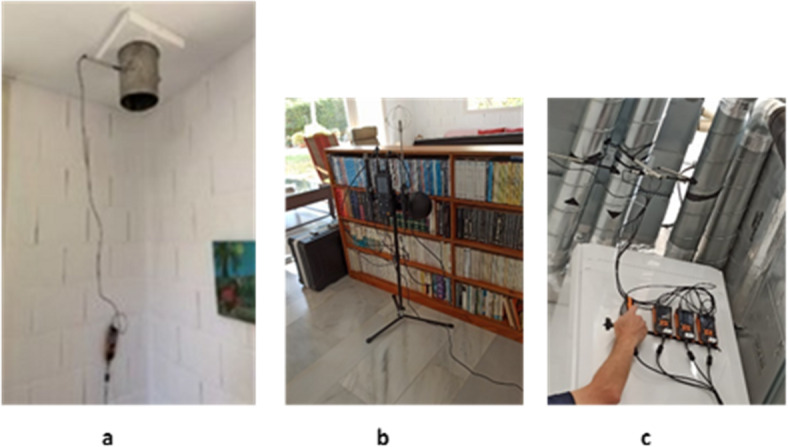
**a** Testo 440 used in the chimneys^29^. **b** Testo 480 used for measuring CO2 and the indoor air temperature during both campaigns. **c** Testo 440 used for measuring the temperature of the mechanical ventilation system.

Figure 7 shows a floor plan view of the dwelling with the different measurement points and sensors used. Figure 8a, 8b and 8c show photographs of the sensors used and Table 5 provides information about the accuracy of the sensors and a description about the measurements.

**Table 5:** Sensors information.

Testo 440	Type	Range	Accuracy	Measurement description
Dry-bulb temperature	NTC	−20–70ºC	± 0.5ºC	To measure the air entering the chimneys, the sensor was installed directly on the vents, at a height of 2.5 m above the floor. For the measurements on the mechanical ventilation system, the sensors were fitted to the inside of the pipes.
Air velocity	Hot wire	0–30 m/s	± (0.03 m/s + 4% of m.v.)	
**Testo 480**	**Type**	**Range**	**Accuracy**	**Measurement description**
Indoor dry-bulb temperature	NTC	0–50ºC	± 0.3ºC	Installed at a height of 1 m above the floor.
CO_2_	-	0–10,000 ppm	± (75 ppm + 3% of m.v.)	
**PCE-FWS 20 N**	**Type**	**Range**	**Accuracy**	**Measurement description**
Outdoor dry-bulb temperature	NTC	−40–60ºC	± 1ºC	Installed on the roof of the dwelling, away from any surface. The point is marked outside the dwelling in Fig. 7 to avoid confusion.
Wind speed	Anemometer	0–50 m/s	v < 5 m/s → ± 1 m/s v > 5 m/s → ± 10%	
Wind direction	Wind vane	-	-	

#### Indoor conditions

**Fig. 9:**
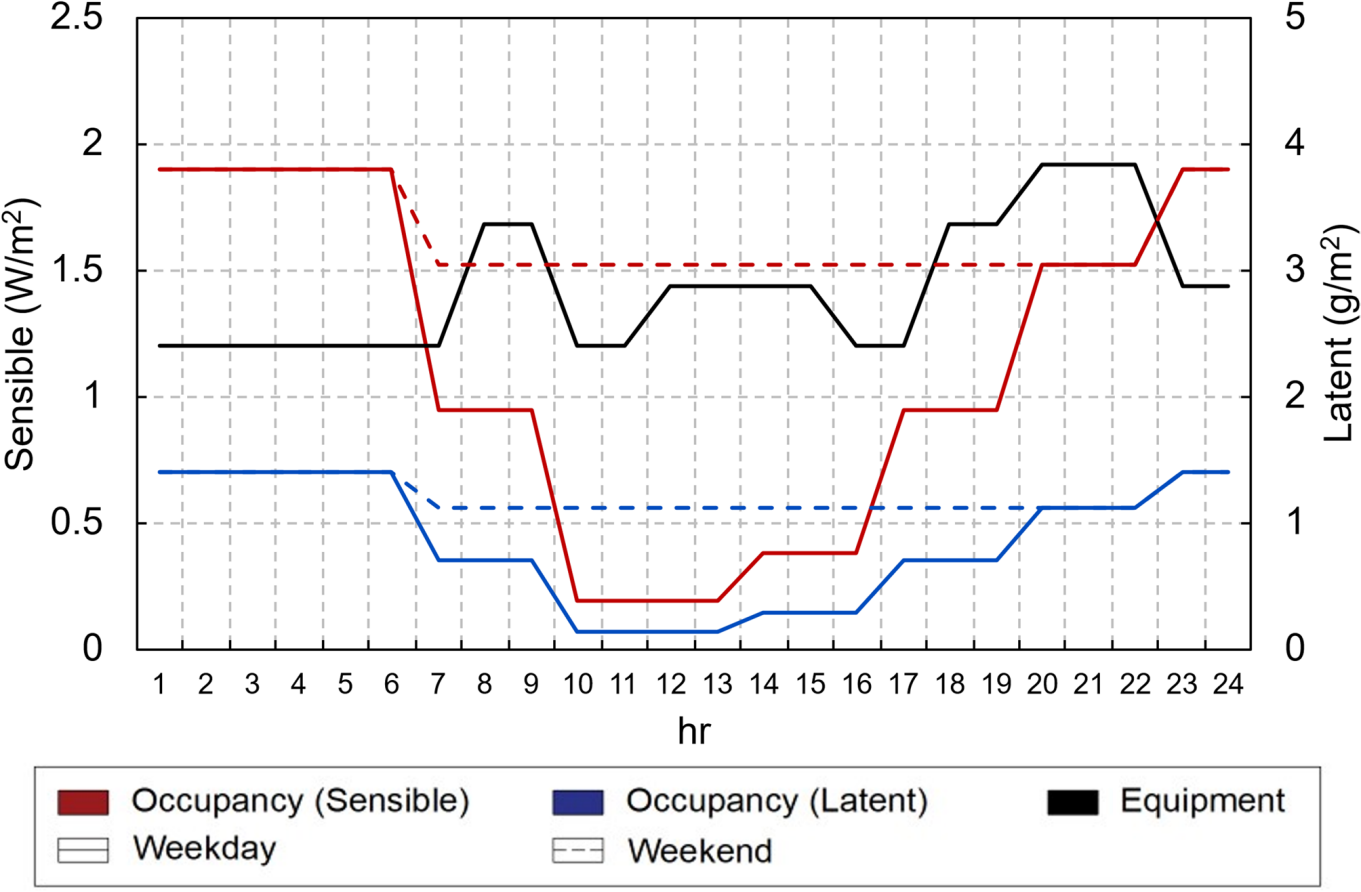
UNE EN 16798-1 user profile.

With respect to the internal thermal loads and indoor comfort conditions, the residential use profile for single-family dwellings proposed in UNE EN 16798-1^32^ was employed. This profile has internal gains of 2.8 W/m^2^ due to occupancy and 2.4 W/m^2^ associated with equipment. Figure 9 shows a graph with the hourly profile and each internal source. Weekdays are shown as a solid line, and weekends are shown as a dashed line. The equipment profile is the same for weekdays and weekends.

This study focused on free-floating conditions so that ventilation is the only system with the possibility of maintaining indoor thermal comfort in summer.

### Climate

**Fig. 10:**
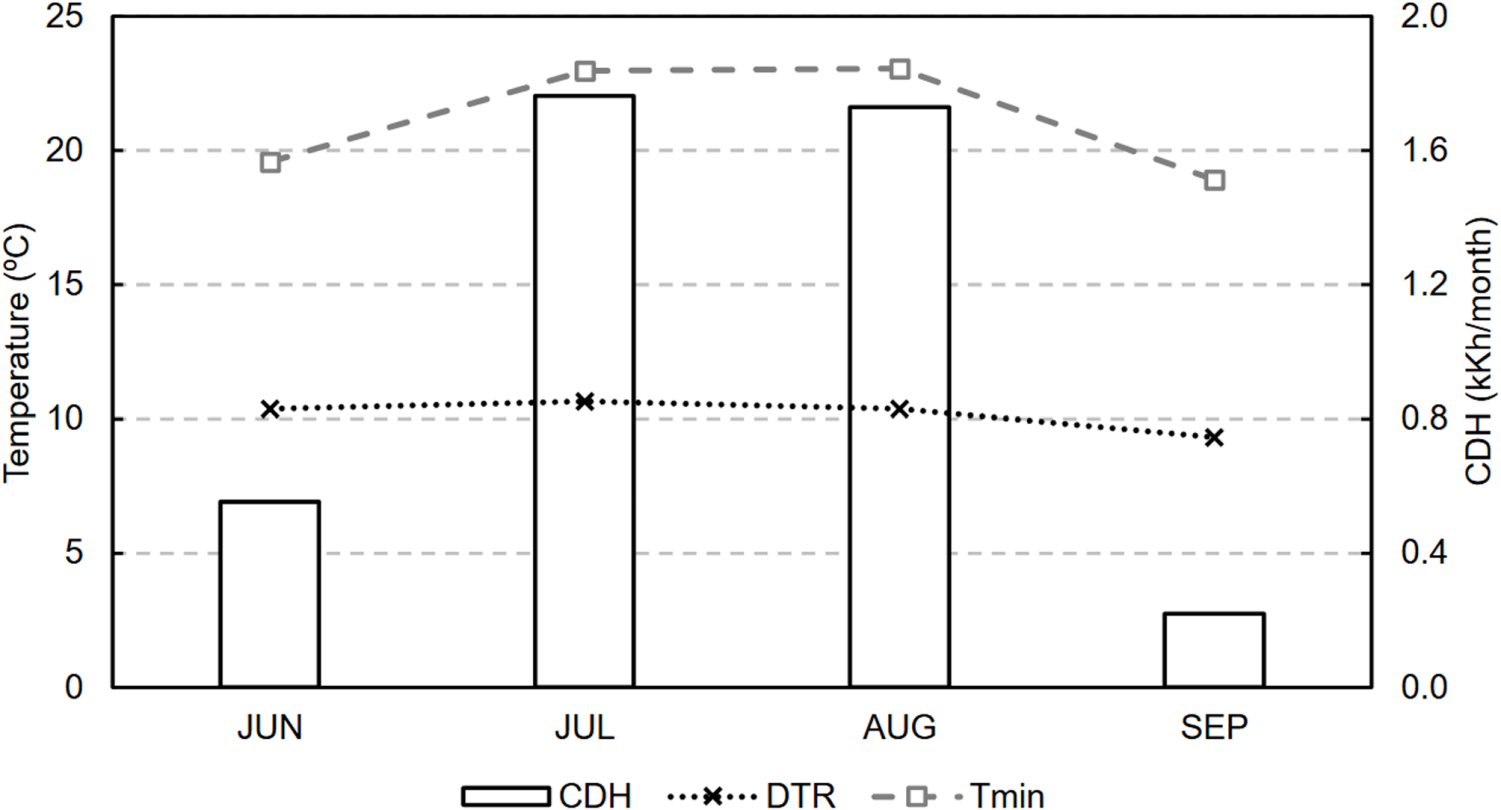
Climate indicators for Los Valientes.

The climate file represents a typical meteorological year (TMY) obtained from the Meteonorm database. In the initial climate analysis, the following metrics are used: the cooling degree hours (CDH) as an indicator of the need for cooling and the mean minimum temperature (T_min_) and the daily amplitude range (DTR) as indicators of the nighttime cooling potential. Figure 10 shows these indicators. The period of greatest interest for both climate analysis and the identification of the most suitable strategy is summer. For this purpose, from now on, the months of interest were established with reference to UNE 100,001^33^, i.e., from June to September (both inclusive).

In Los Valientes, July and August by far exhibit the highest cooling demands (from now on, the warmer months). The DTR remains practically constant because of the proximity of Los Valientes to the Mediterranean coast. During the cooling period, the DTR is approximately 10 ºC, and the annual average is 9.8 ºC. The other indicator, T_min_, increases together with the CDH and reaches its highest values in the warmer months, with a value of approximately 23 °C. In the remaining summer months, T_min_ is below 20 °C.

### Case studies

Three free-floating scenarios for summer were considered according to the ventilation system:

- NV: Natural ventilation scenario. This is the current scenario used by the occupants of the house. Ventilation is achieved naturally through a ventilation system, which includes solar chimneys and a wind tower. During summer nights, windows in the southern façade are opened from 9 p.m. to 9 a.m., thereby promoting greater ventilation airflow.
- MHRV: Mechanical ventilation scenario. Given the implementation of the MHRV system, a scenario in which the dwelling is mechanically ventilated was proposed. Under this scenario, the natural ventilation system is switched off. During this scenario, the dwelling has a constant ventilation rate of 130 m^3^/h with heat recovery and the possibility of bypass if outdoor conditions allow. No windows were opened during this scenario.
- NV + MHRV: Preliminary analysis of the individual scenarios showed that a third combined scenario had the advantages of both individual scenarios together. For this reason, this third scenario was proposed, where mechanical ventilation was used during the day and natural ventilation at night. This scenario does not present simultaneity of systems, but for the same day each system was activated in a different schedule.

### Metrics for assessing ventilation performance

#### Indoor air quality

Since ventilation aims to ensure IAQ conditions, a parameter was defined to identify the performance of natural ventilation. This ratio is the quotient of the number of hours in which the number of ACH is greater than the minimum value (that ensures IAQ), divided by the total number of hours in each month Eq. (4). The minimum ACH value for La Casa de la Tierra is 0.31 h^−1^, defined according to the CTE DB-HS/3.

4 $$\:r=\frac{\sum\:_{month}\gamma\:\left(ACH\left(h\right)\right)}{\sum\:_{month}h}$$

5 $$\:\gamma\:\left(ACH\left(h\right)\right)=\left\{\begin{array}{c}1,\:\:ACH\left(h\right)\ge\:AC{H}_{min}\\\:0,\:\:ACH\left(h\right)<AC{H}_{min}\end{array}\right.$$

where:

- $\:ACH$: the number of air change rate per hour;
- $\:h$: the time of the month; and
- $\:AC{H}_{min}$ the minimum ACH value to achieve the IAQ.

#### Sensible heat load and energy consumption

The sensible ventilation heat load is formulated in the TRNSYS zonal energy balance (NTYPE904) using NTYPE954 for infiltration and NTYPE955 for ventilation, both of which are formulated using Eq. (6).

6 $$\:{\dot{Q}}_{vent,sens}=\dot{\nu\:}\rho\:{c}_{p}\left({T}_{air}-T\right)$$

where:

- $\:{\dot{Q}}_{vent,sens}$: the sensible heat load from ventilation or infiltration;
- $\:\dot{\nu\:}$: the volumetric airflow rate of ventilation or infiltration;
- $\:{c}_{p}$: the specific heat of air;
- $\:{T}_{air}$: the temperature of ventilation or infiltration air; and
- $\:T$: the air temperature at the node.

For each day, there will be times when the sensible heat load is either positive or negative, with the latter being beneficial as ventilation then helps to reduce the cooling demand. Depending on the ventilation system, T_air_ will be either directly the outdoor air temperature or the supply temperature of the heat recovery unit.

As the sensible heat load due to ventilation must be addressed by cooling equipment, this energy consumption was estimated under the assumption of a vapour compression system with a constant SEER value. A SEER value of 3.5 was set for the cooling equipment, and for the energy consumption associated with the heat recovery unit, its SFP (defined in Table 2) was used directly. The final energy consumption for each strategy was associated with the ventilation system, i.e., the NV strategy only represents the energy consumption associated with the sensible ventilation thermal load, whereas the MHRV strategy represents the consumption associated with the sensible ventilation thermal load and the energy consumption of the fans.

In TRNSYS, the simulation timestep was set to 1 h. Thus, the sum of the sensible heat loads during all hours of the month is the cooling sensible demand associated with that sensible heat load. Therefore, the final energy consumption (FEC) due to ventilation is defined for each month and system according to Eqs. (7)-(9).

7 $$\:{FEC}_{NV}=\frac{\sum\:_{month}{\dot{Q}}_{vent,sens}}{SEER}\:$$

8 $$\:{FEC}_{MHRV}=\frac{\sum\:_{month}{\dot{Q}}_{vent,sens}}{SEER}+\sum\:_{month}{\dot{P}}_{fan}$$

9 $$\:{FEC}_{NV+MHRV}=\frac{\sum\:_{month}{\dot{Q}}_{vent,sens}}{SEER}+\sum\:_{month}{\dot{P}}_{fan}$$

where:

- $\:{FEC}_{NV}$: the final energy consumption due to the sensible ventilation heat load under the natural ventilation strategy; and
- $\:{FEC}_{MHRV}$: the final energy consumption due to the sensible heat load of mechanical ventilation considering the energy consumption of the fans.
- $\:{FEC}_{NV+MHRV}$: the final energy consumption due to the sensible heat load under the combined strategy considering the energy consumption of the fans.

**Fig. 11:**
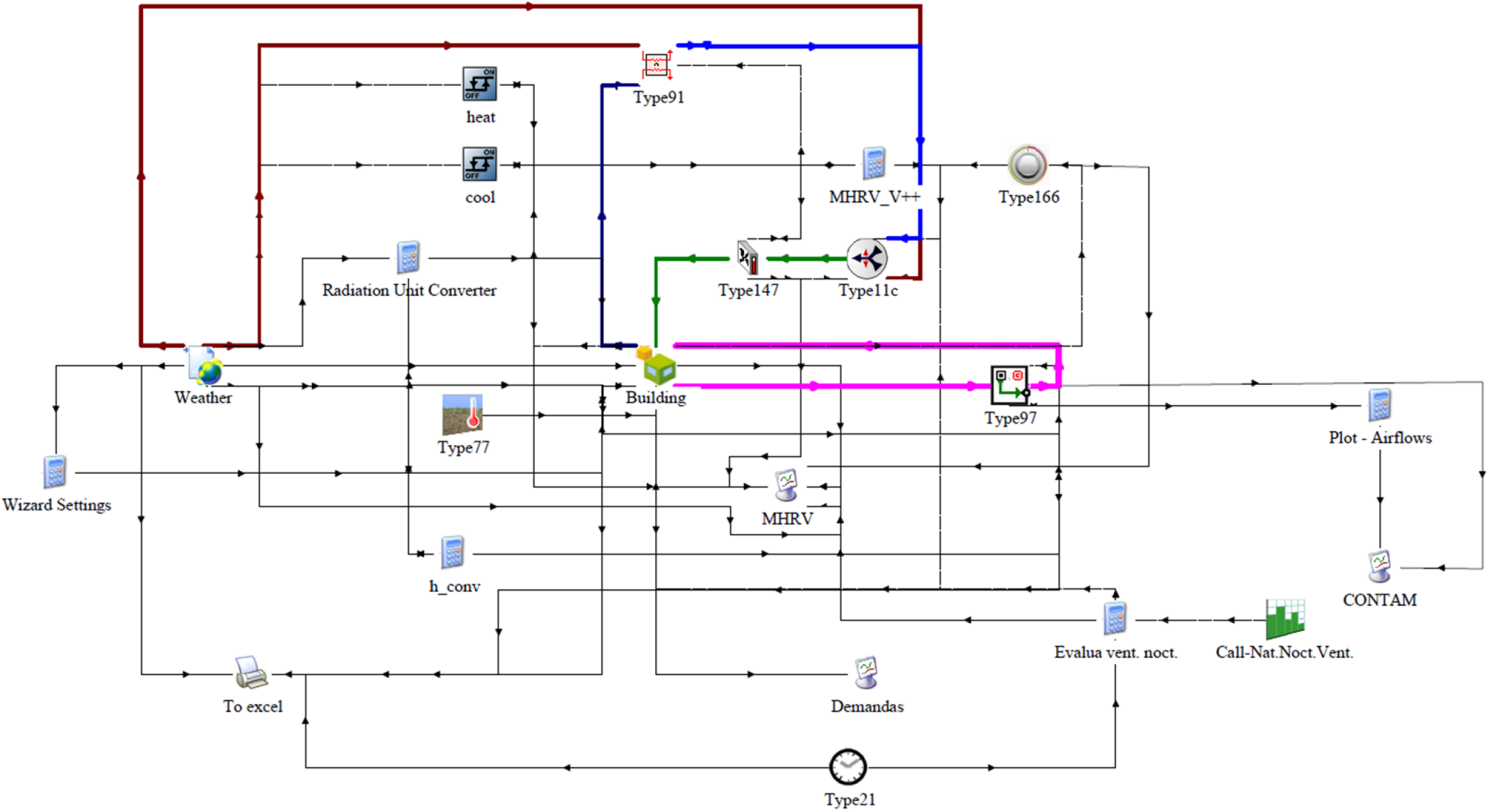
Simulation scheme developed in TRNSYS.

Finally, Fig. 11 shows the simulation scheme developed in TRNSYS^27^where the nighttime ventilation period was set by schedules (Type14h).

## Results

### Model validation

**Fig. 12:**
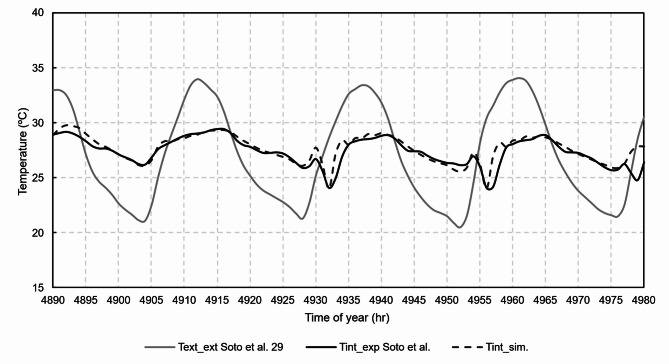
Comparison of the experimental and simulated temperatures.

**Fig. 13:**
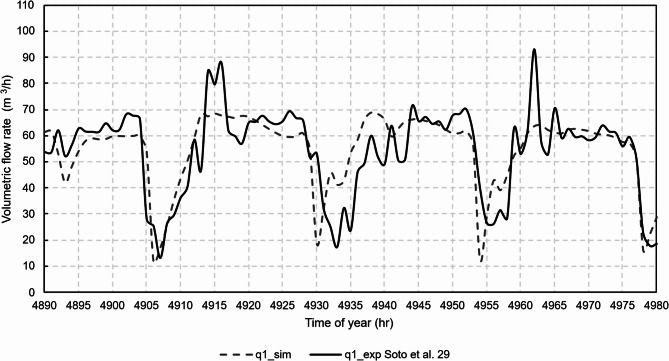
Comparison of airflow rates, chimney 1.

**Fig. 14:**
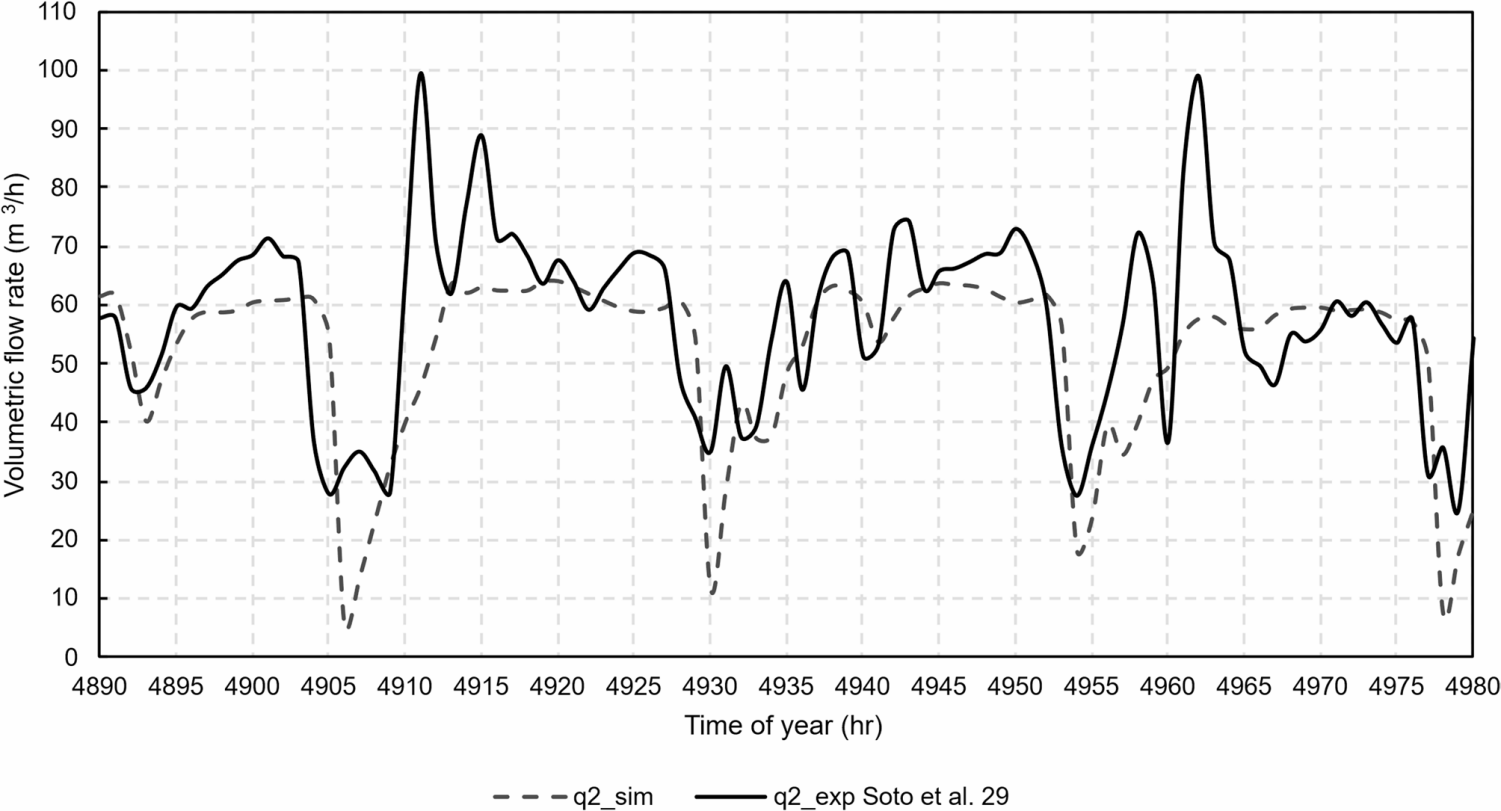
Comparison of airflow rates, chimney 2.

**Fig. 15:**
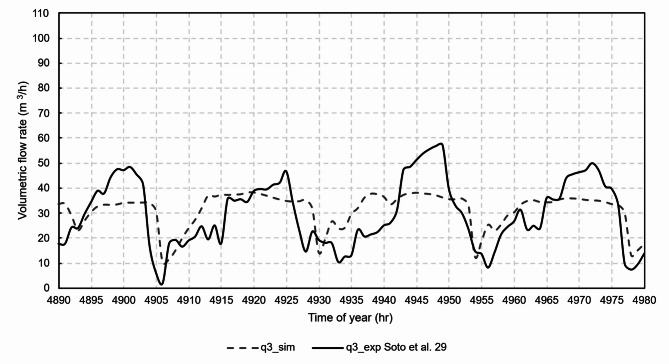
Comparison of airflow rates, chimney 3.

**Fig. 16:**
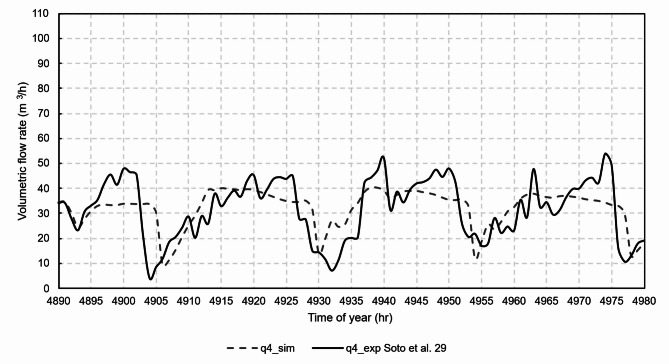
Comparison of airflow rates, chimney 4.

Figures 12, 13, 14, 15 and 16 show graphical comparisons between the indoor temperature and the ventilation airflow rates estimated by the new model and experimentally.

**Fig. 17:**
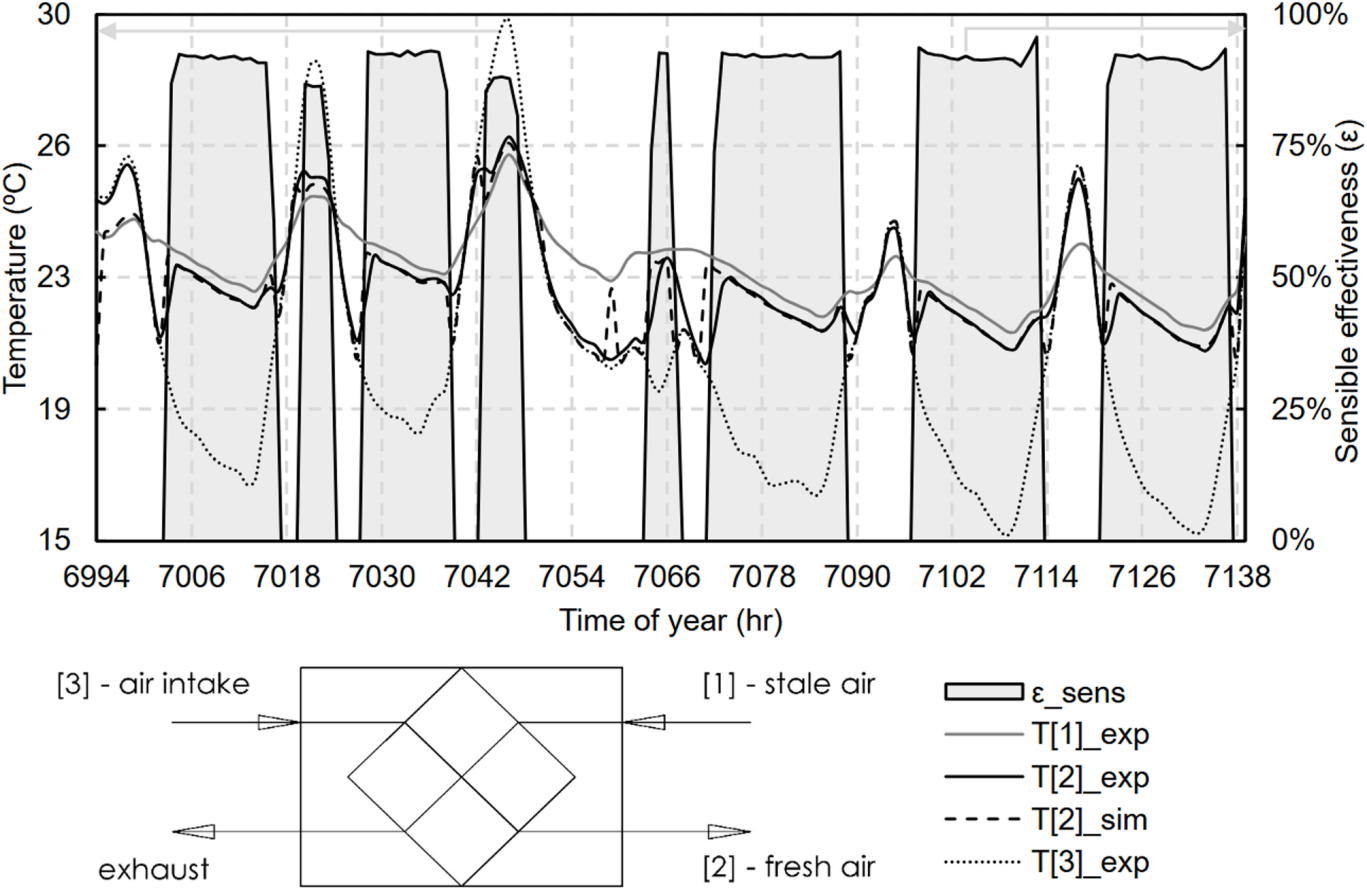
Comparison of the fresh air outlet temperatures for the MHRV system.

With respect to the mechanical system, temperatures were measured at the exterior air intake, fresh air supply outlet and stale air inlet to assess the bypass operation. Figure 17 shows these results together with the sensible effectiveness value. In the model, the system was configured so that it bypasses the heat exchanger when the indoor–outdoor temperature difference is at least 1 K.

During the experimental campaign, a total of four occupants conducted normal living in the dwelling, the occupancy being variable during the weekdays, and complete during the weekends. The thermal model responded satisfactorily using the fixed use profile of the EN 16798-1 standard, with an RMSE between the experimental and the predicted by the model of 0.7 ºC, the same result was obtained for the supply temperature of the mechanical ventilation system.

For the natural ventilation model, the RMSE between experimental and model was 11.1 m^3^/h for the airflow rate of chimney 1, 14.5 m^3^/h for the airflow rate of chimney 2, 10.6 m^3^/h for the airflow rate of chimney 3 and 9.2 m^3^/h for the airflow rate of chimney 4. Once the model was experimentally validated, the case studies were then analysed.

### Indoor air quality

To assess the ability of natural ventilation to maintain the IAQ, the *r*(ACH) metric defined in Sect. Indoor air quality was used. Table 6 shows the results for all summer months and with a distinction between day and night.

**Table 6:** Percentage of hours in which natural ventilation ensured a minimum airflow rate.

Month	Daytime				Nighttime			
	*r*(ACH)	ACH_max_	ACH_mean_	ACH_min_	*r*(ACH)	ACH_max_	ACH_mean_	ACH_min_
Jun	52%	0.44	0.30	0.07	100%	3.84	3.23	2.23
Jul	56%	0.43	0.32	0.17	100%	3.82	3.10	2.25
Aug	54%	0.45	0.30	0.13	100%	3.73	2.99	2.39
Sep	52%	0.53	0.32	0.07	100%	3.85	3.33	2.63

In summer, during the day, although the average ACH value was slightly below the normative minimum, *r*(ACH) values slightly above 50% of the total hours were reached. In contrast, at night, by allowing a greater airflow by opening windows in the southern façade, the ventilation efficiency reached 100%. This was due to the fact that the average value was around 3 ACH with maximums close to 4 ACH.

**Fig. 18:**
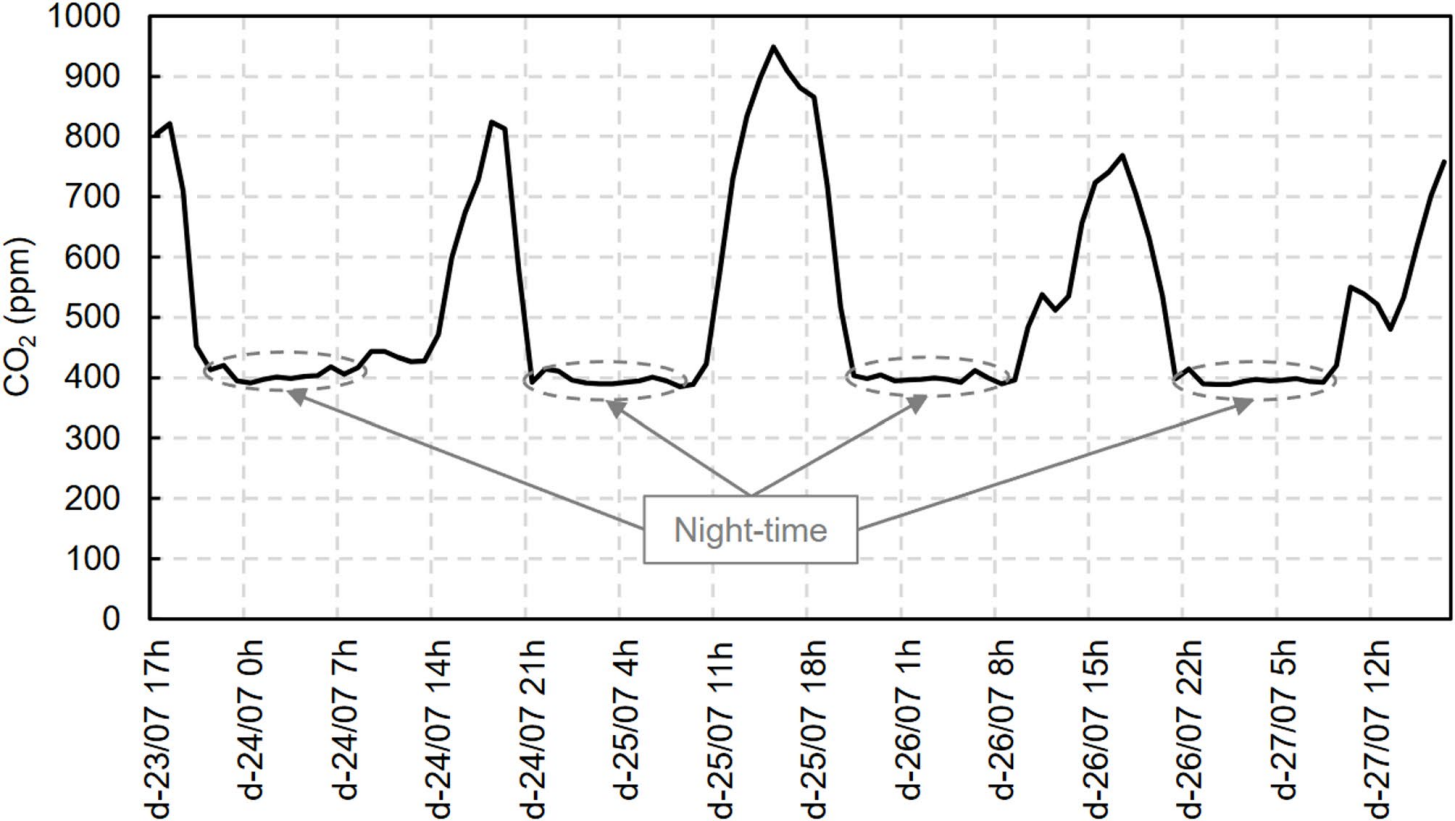
CO_2_ level in the dwelling. Experimental data^29^.

During data collection, the measured CO_2_ levels were consistent with the results of the *r*(ACH) metric. Figure 18 shows the measured CO_2_ levels (during the NV scenario only). During the day, peaks of 800 ppm and in one case higher than 900 ppm were reached, consistent with the low *r*(ACH) value of 58% for the same period. At night, the CO_2_ levels decreased to the lowest values observed, approximately 400 ppm. The model presented an *r*(ACH) of 98% for these same nights.

### Sensible heat load

**Fig. 19:**
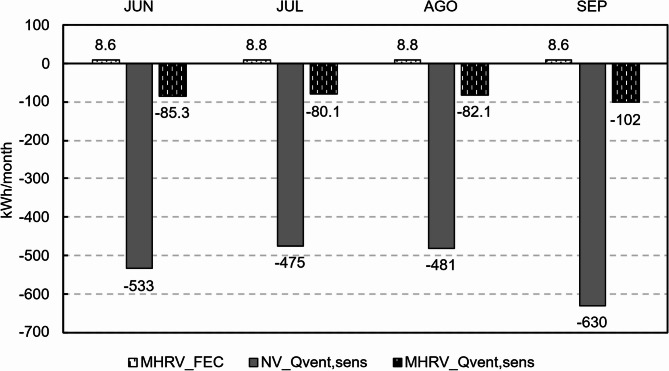
Sensible thermal load due to ventilation at night.

**Fig. 20:**
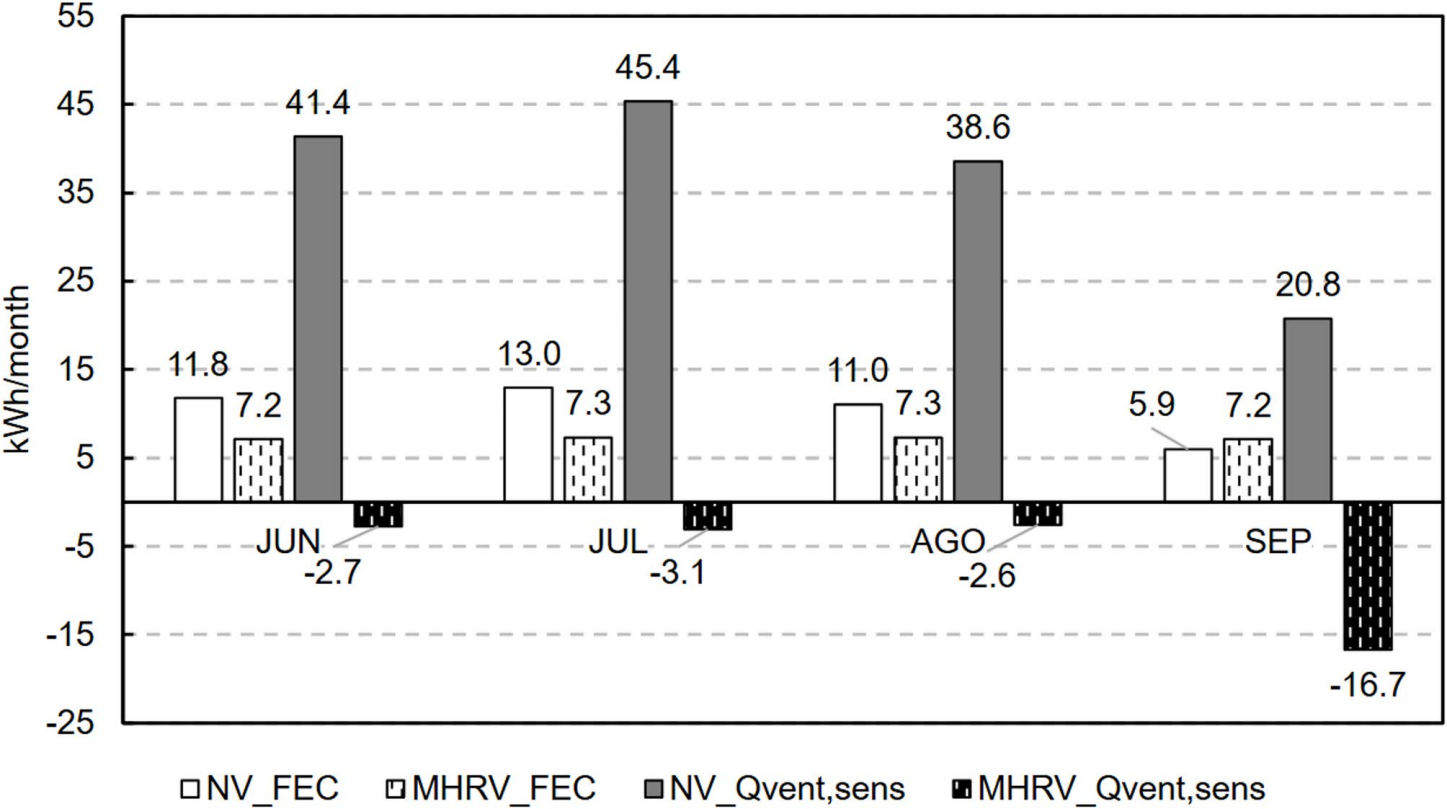
Sensible thermal load and final energy consumption due to ventilation during the day.

Figures 19 and 20 show the sensible thermal heat load and energy consumption, respectively, due to ventilation (the metrics are defined in Sect. Sensible heat load and energy consumption) for each strategy. As done for the IAQ analysis, a distinction is made between nighttime and daytime. The darker colours represent the sensible thermal load (Q_vent, sens_), and the lighter colours denote the final energy consumption (FEC).

At night (Fig. 19), owing to the lower outdoor temperatures, both systems exhibited a negative sensible thermal load, with natural ventilation offering the best performance because of the higher airflow introduced. Despite providing an automatic bypass system, the mechanical system was could not utilise the full cooling potential available because of its lower ventilation flow rate. When the energy consumption was evaluated, only the MHRV scenario exhibited energy consumption at night because of the fans. This consumption was similar across all months, with an average value of 8.7 kWh per month.

In contrast, during the day (Fig. 20), the sensible ventilation heat load was mostly positive when natural ventilation was used, whereas when the mechanical system was employed to recover heat and allow bypass, the sensible ventilation heat load was negative. However, this did not exclusively occur because the outside air exhibited a temperature within the comfortable region (desired situation for bypass) but rather because the mechanical system maintained a higher indoor temperature with a lower amplitude than did natural ventilation. A detailed analysis is provided in Sect. Thermal comfort.

### Thermal comfort

**Fig. 21:**
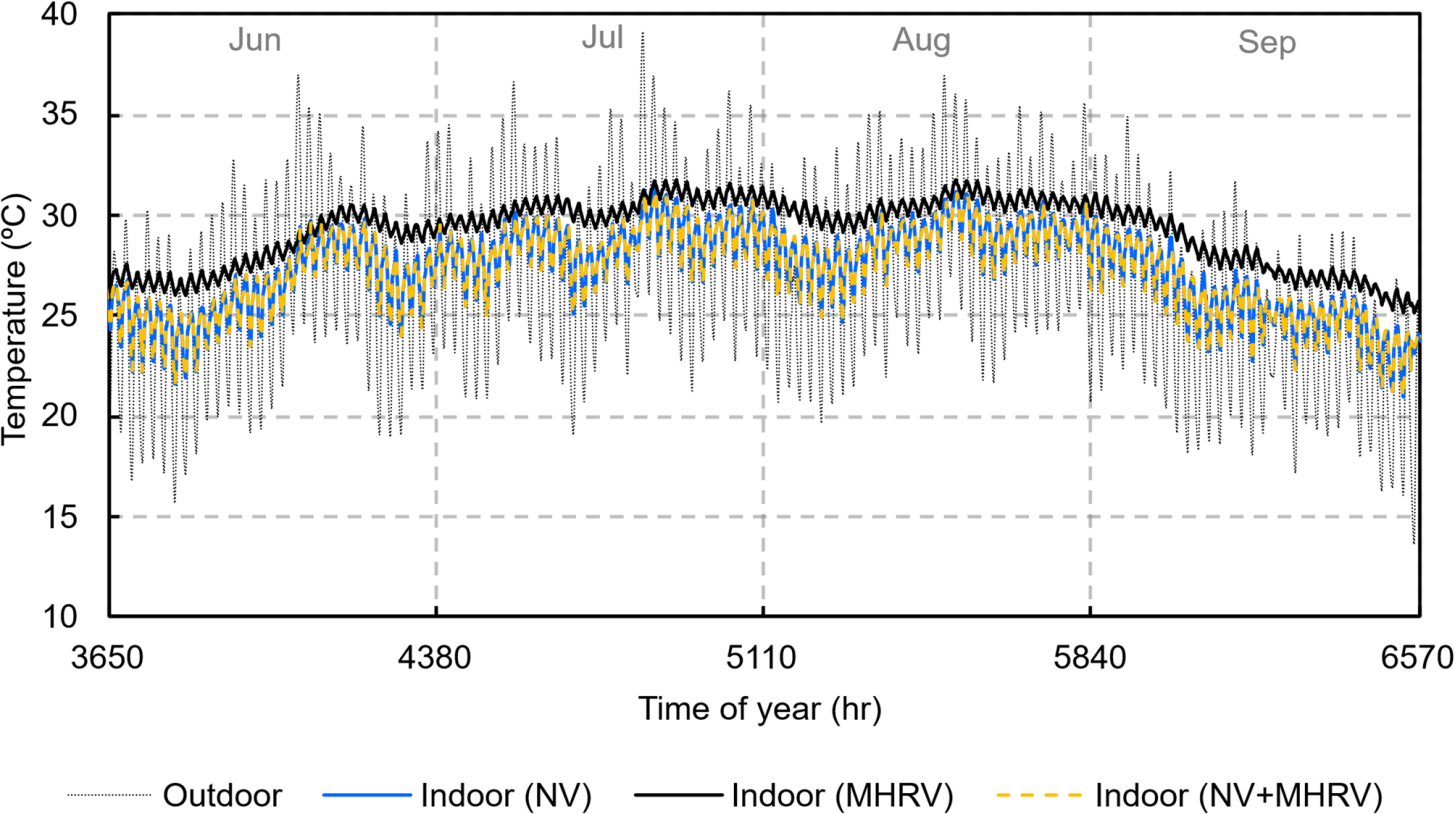
Temperature evolution for all scenarios.

Figure 21 shows the temperature evolution for all scenarios. Due to the lower ventilation airflow, the MHRV scenario at night did not dissipate enough heat, causing a heat trap effect that caused the indoor temperature of the dwelling to stabilise at a higher value the next day. If a criterion of 26 °C is set for the maximum comfort temperature, only natural ventilation could keep the dwelling below this limit for a short period of time, despite a positive sensible ventilation heat load during the day. These periods corresponded mainly to the less hot months (June and September).

However, the MHRV scenario was the only one that made it possible to ensure IAQ during days and nights, which was only possible during nights with the NV scenario. These results led to the proposal of a third scenario in which ventilation was provided during the day by a mechanical ventilation system and at night by natural means, where both modalities can ensure the IAQ and maintain lower indoor temperatures.

**Fig. 22:**
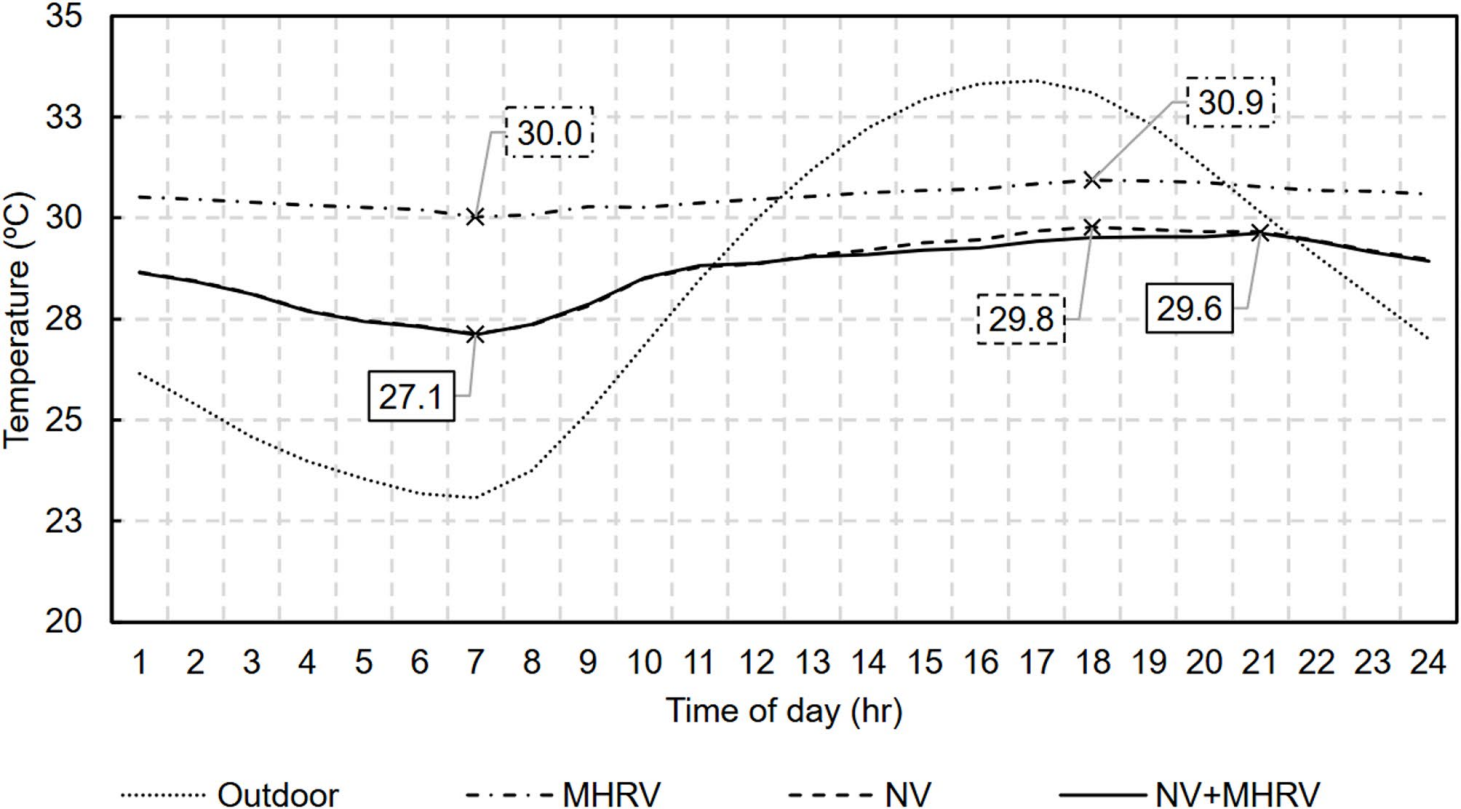
Average indoor temperature in the month of August.

Figure 21 also shows the indoor temperature evolution for this third scenario, which was similar to that of the NV scenario. For a more detailed analysis, Fig. 22 shows the average indoor temperature during the hottest summer month (August) for the previous scenarios and the third proposed scenario considered in this work. The effect of natural ventilation at night is observed as the average indoor temperature decreases to 2.5 K below the daily average. The MHRV during the day helps maintain a lower temperature than does the MHRV-only strategy, where the daily average oscillates above 30 °C.

This combined strategy also helped reduce the maximum indoor temperature. In August, the maximum indoor temperatures were 31.1 °C, 31.2 °C, and 31.8 °C for the NV + MHRV, NV, and MHRV scenarios, respectively. The largest difference was observed with respect to the MHRV-only strategy and reached 0.7 K. This difference, although seemingly negligible, helps to reduce the peak cooling demand.

In terms of unmet hours (UH), Table 7 shows, by month, the UH for each scenario. Of the three scenarios, the two scenarios where night ventilation was provided by natural means had the lowest UH, with the largest differences observed in the less severe summer months of June and September. For these months, the lack of comfort was reduced by more than 50% compared to the MHRV scenario.

**Table 7:** Unmet hours.

Month	NV	MHRV	NV + MHRV
June	34%	68%	33%
July	87%	100%	88%
August	91%	100%	91%
September	24%	67%	23%

### Final energy consumption due to ventilation

**Fig. 23:**
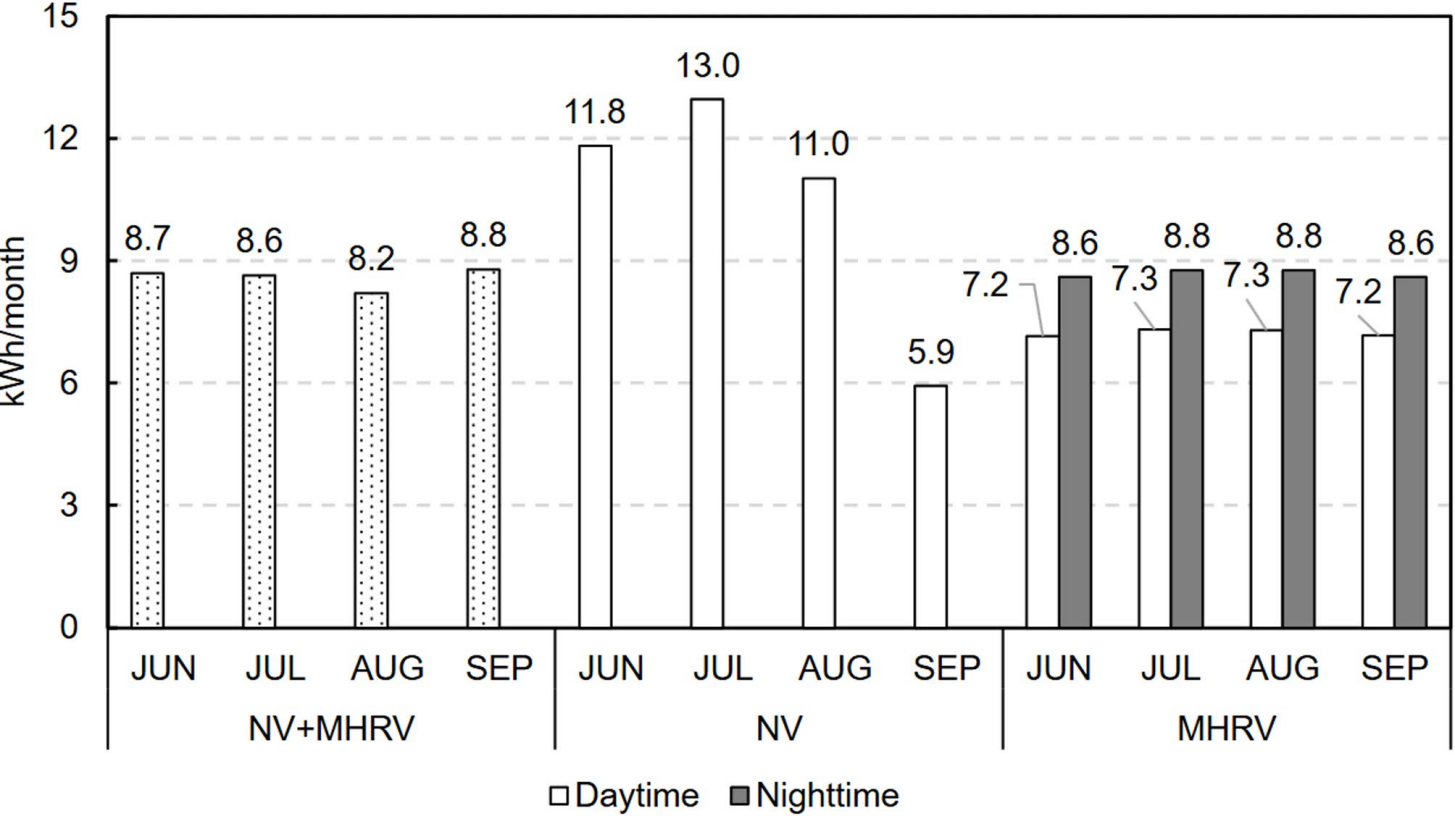
Final energy consumption results for all the scenarios.

The conclusions derived from the analysis from the perspective of the final energy consumption (FEC) are the same. Notably, the use of the combined strategy optimises the energy consumption of both scenarios. Figure 23 shows the final energy consumption (FEC) for the optimal strategy and the previous scenarios.

The FEC due to ventilation was 0.23 kWh/m^2^-y for the NV + MHRV scenario, the lowest of the proposed scenarios, compared with the values of 0.28 kWh/m^2^-y for the NV-only scenario and 0.42 kWh/m^2^-y for the MHRV-only scenario.

## Discussion

Among the scenarios presented here with a single system, only the MHRV scenario satisfied the health requirements, making it valid according to Spanish regulations. This ventilation strategy allows to take advantage of night ventilation, by bypassing the intake air when outdoor conditions were favourable, which provided some cooling to the dwelling. However, it was more favourable to combine mechanical ventilation during daytime hours and switch to natural ventilation at night, both in terms of energy consumption and night-time cooling. It was demonstrated that the NV + MHRV scenario, namely, a combination of both scenarios, also ensured the IAQ during all summer hours. The latter is important since otherwise it would not be possible to implement this strategy in Spain and would be limited to a scenario with continuous mechanical ventilation.

On the one hand, this scenario, by ensuring natural ventilation during summer nights, eliminated the energy consumption of the fans, resulting in a 46.2% reduction in ventilation energy consumption in summer. On the other hand, by ventilating with higher ventilation rates, NV + MHRV yielded an approximately 6.3 times greater nighttime cooling potential than did the MHRV-only scenario, achieving a total of 14.2 kWh/m^2^-y, similar to the average value estimated by Santamouris et al.^16^. The latter allowed for a reduction in UH in June and September by 52% and 66% respectively compared to the MHRV scenario.

La Casa de la Tierra, despite being conceived as bioclimatic, presents ventilation requirements to ensure IAQ consistent with that of a typical single-family dwelling in Spain and for some European countries. The latter makes it possible to generalise these results to single-family dwellings in other regions. On the one hand, Artmann et al.^12^ demonstrated that northern Spain encompasses regions with potentials up to twice as high as those in southern Spain. On the other hand, according to Campaniço et al.^14^the same region (Los Valientes) exhibits potential, at approximately 0.5–1 kWh/m³ in the summer months, while regions further north reaching up to 3 kWh/m³ for ventilation rates of 1.5 ACHs. For comparison, Table 8 provides the cooling potential by ventilation estimated by these authors and those obtained in this study in terms of energy per unit of usable floor area of the dwelling (kWh/m^2^) for the summer months. For the conversion of ACH, the average of all months was chosen, i.e., 3 ACHs.

**Table 8:** Comparison of cooling potential results.

Month	Cooling potential (kWh/m^2^-month)	
	Campaniço et al.^14^	Present study
June	7.5–9.2	3.6
July	3.8–4.6	3.2
August	3.8–4.6	3.2
September	7.7–9.1	4.2

In the work of Campaniço et al.^14^ventilation was allowed whenever the outside air exhibited a lower temperature than the building. This explains the large differences observed in the transitional or less severe summer months (June and September), when this strategy provides greater cooling potential. In this study, a nighttime ventilation schedule between 9 p.m. and 9 a.m. was maintained throughout all summer months, indicating that during these less severe months, the nighttime ventilation schedule should be extended to increase the effectiveness of nighttime cooling.

The above is observed in the more severe summer months, where the differences are smaller. As nights are hotter, the schedule used in this study prevents wasting the cooling potential available at other times. However, there is still no temperature control, which leads to ventilation with air under unfavourable conditions, hence the observed differences. To fully utilise the cooling potential, if this strategy is to be used in other regions, the natural ventilation time slot must be adapted. Regions with higher cooling potential will have more hours where the outside temperature is below the maximum required for comfort.

### Limitations and future work

The impact of uncontrolled air flows, i.e. lack of permeability, was not considered. A leaky building will experience a higher rate of ventilation by natural means. During summer nights this is beneficial, as the total amount of free cooling will be higher. However, during the days and with the MHRV system (proposed daytime system in this work), infiltrations will mean that there is an amount of air entering the building untreated and therefore it will be at the outdoor temperature. This reduces the overall heat recovery effectiveness with a consequent additional positive heat load.

Finally, this study was conducted under freefloating conditions. The next step will be to implement a cooling system and study its effect on the proposed strategy.

## Conclusions

The aim was to study the applicability of ventilation as a cooling technique exploring the possibility of doing it by mechanical means or by natural means and ensuring IAQ requirements. To this end, an energy model of a detached single-family dwelling (La Casa de la Tierra) was developed, coupled with a natural ventilation model and a mechanical ventilation unit with heat recovery. The model was validated experimentally, and ventilation strategies were studied. The conclusions of this work are:

1. Of single-system scenarios, only the mechanical ventilation with heat recovery (MHRV) met the indoor air quality requirements established by the Spanish regulations. However, the combination of natural ventilation during nighttime with MHRV during daytime (NV + MHRV), also guaranteed IAQ throughout the summer, which validates its application in the IAQ regulatory context.
2. The NV + MHRV scenario proved to be more energy efficient, reducing ventilation energy consumption by 46.2% during summer by eliminating the use of fans at night. In addition, this scenario offered a night cooling potential 6.3 times higher than the MHRV scenario.
3. Thanks to the higher cooling potential, the NV + MHRV scenario was able to reduce unmet hours in June and September by 52% and 66% respectively, compared to the MHRV scenario.

## Data Availability

The datasets generated during the current study are available from the corresponding author on reasonable request.
